# Modular Construction of Multivariate Metal–Organic Frameworks for Luminescent Sensing

**DOI:** 10.1021/jacs.4c17248

**Published:** 2025-01-15

**Authors:** Zongsu Han, Kun-Yu Wang, Rong-Ran Liang, Yifan Guo, Yihao Yang, Mengmeng Wang, Yue Mao, Jiatong Huo, Wei Shi, Hong-Cai Zhou

**Affiliations:** † Department of Chemistry, Texas A&M University, College Station, Texas 77843, United States; ‡ Health Science Platform, Tianjin University, A203, Bldg. 24, 92 Weijin Rd., Nankai Dist, Tianjin 300072, China; § Frontiers Science Center for New Organic Matter, State Key Laboratory of Advanced Chemical Power Sources, and Key Laboratory of Advanced Energy Materials Chemistry (MOE), College of Chemistry, Nankai University, Tianjin 300071, China

## Abstract

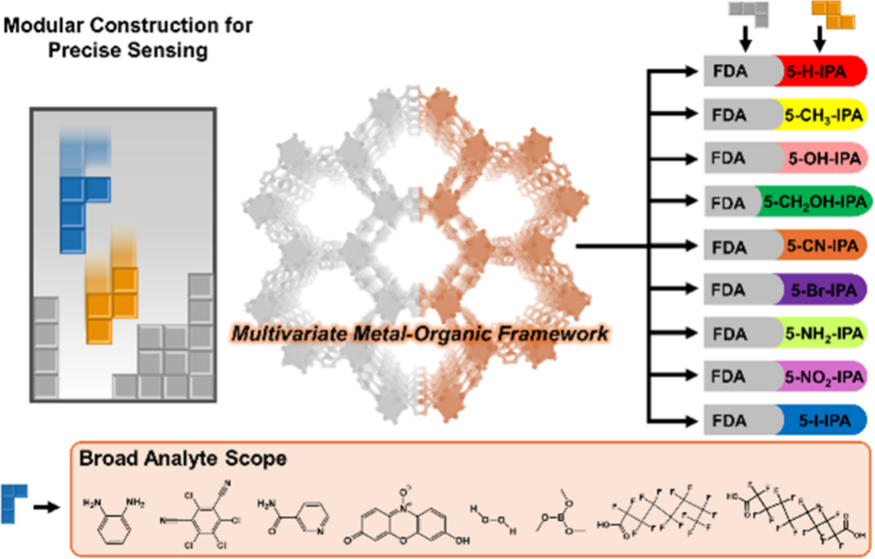

Metal–organic frameworks (MOFs) have played a pivotal role as rapid and effective luminescent sensing materials. Numerous MOFs with diverse characteristics have been meticulously designed for target analytes. Previous studies have highlighted the factors of spectral characteristics, energy levels, interaction forces, and sensor stabilities for the luminescent sensing performance in response to a specific analyte. This conventional “point-to-point” approach necessitates the matching of sensing materials to a specific analyte. Herein, we develop a modular MOF-based luminescent sensing platform by using a mixed-ligand strategy. A luminescent MOF Eu-FDA with 2,5-furandicarboxylic acid can serve as the foundational platform, with partial replacement by nine distinct hexacyclic isophthalic acids as the modules, respectively, to specifically accommodate different analytes with particular structures and properties. This substitution has been meticulously confirmed through single-crystal X-ray diffraction. Confronted with analytes possessing diverse structural or property characteristics, modular isophthalic acid derivatives can enhance the sensing capability to achieve heightened sensitivity.

## Introduction

Rapid development of modern society is accompanied by the exponential growth of the global chemical market and the discovery of numerous new compounds every year, which have widespread applications as industrial raw materials, medicinal agents, and pesticides;^1−3^ however, certain chemicals have been identified as toxic substances, encompassing carcinogens, teratogens, and lethal compounds.^4−8^ As a result, individuals are exposed to a variety of chemicals in both daily life and production, raising the urgent need for rapid and quantitative recognition. Herein, luminescent sensing has emerged as a highly effective method for detecting various analytes, including heavy metal ions, volatile organic compounds, persistent organic pollutants, health-related medicines, and biomarkers.^9−11^ In comparison to other quantitative analysis techniques, luminescent sensing features unique merits of instant response, high sensitivity, and nondestructive analysis of samples. Furthermore, it can be integrated into compact low-cost devices, meeting the demands for both qualitative visual inspection and high-precision quantitative analysis.^12−14^ Yet, the practical application of luminescent sensing is hindered by its limited substrate scope and interference resistance. In previous studies, researchers have developed various functional sensing materials toward targeting analytes.^9−14^ Nevertheless, the state-of-the-art design of luminescent sensing materials is mostly based on a “trial-and-error” method, wherein one sensor is prepared, corresponding to one specific analyte. Such a research flow not only lacks in the targeted design and synthesis of sensors but also inevitably leads to substantial redundant consumption of time and energy, which may hinder the efficient development of this field.

**Figure 1:**
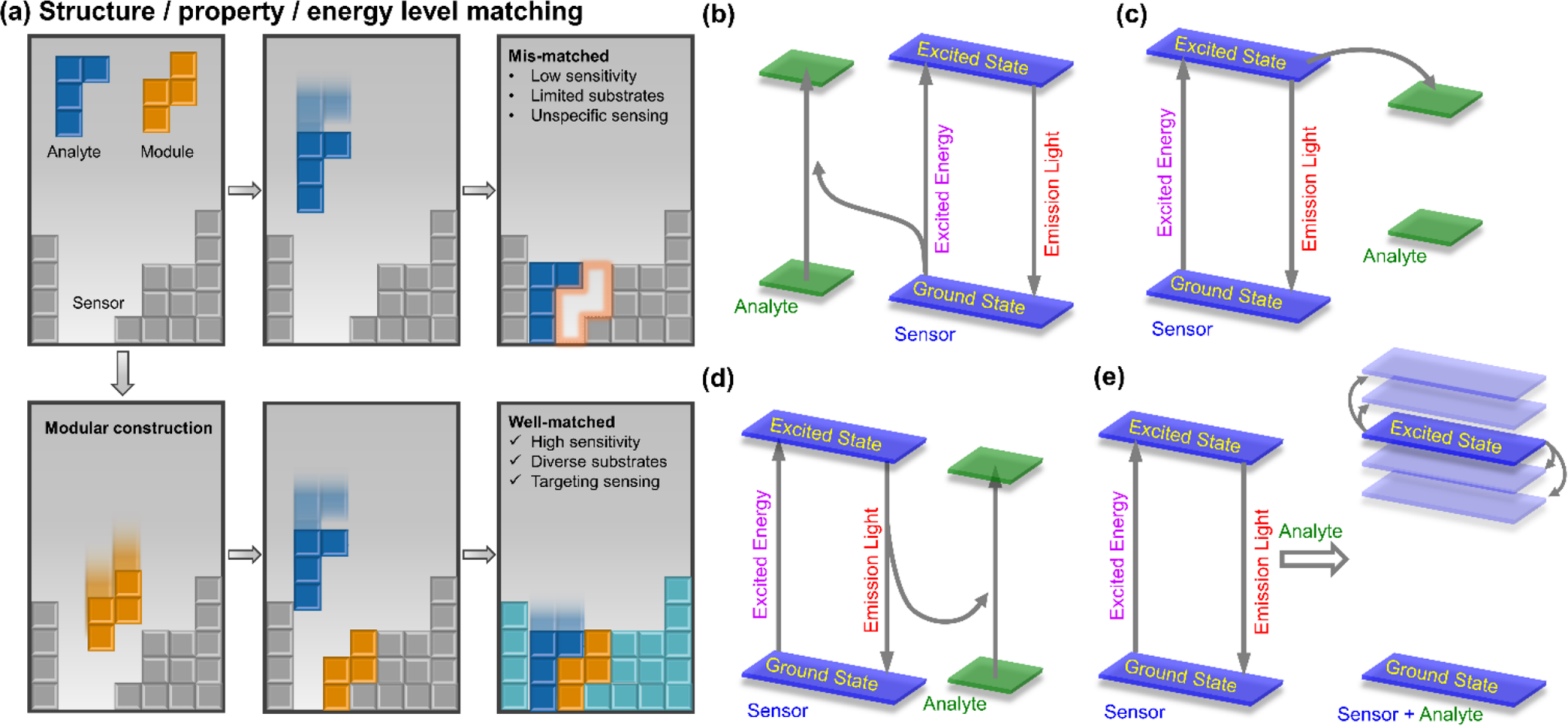
Material design principle and simplified luminescent sensing mechanisms in this work. (a) Modular construction allowing to match modules and analytes based on their characteristics. (b) Competition absorption process. (c) Photoelectron transfer process. (d) Förster resonance energy transfer process. (e) Structural decomposition/transformation process.

To get a general role to develop luminescent sensors for practical applications, we introduce the concept of “modular design” to construct multivariate luminescent sensing materials (Figure 1a).^15−18^ Note that the components within these materials are divisible into distinct modules that can be independently edited or replaced. Aiming at specific analytes, the luminescent modules of sensing materials can be precisely regulated, achieving unprecedented specificity and efficiency in recognition while adapting to the complex working conditions in reality. The advantage of the “modular construction” strategy lies in its combination of reproducibility associated with standardization and high selectivity afforded by customization. This approach not only reduces the “trial-and-error” costs in material preparation but also significantly facilitates the iterative optimization of the materials. A fundamental prerequisite for realizing modular construction is that the materials must possess component slottability, implying that functional modules can be dynamically integrated into the system, ensuring perfect matching with analytes.

Herein, we report the application of metal–organic frameworks (MOFs) with tailored modules as the prototype for achieving luminescent sensing toward multiple analytes. MOFs have been recognized as promising sensing materials owing to their diverse chemical compositions, tailorable pore environment, and crystallographically defined structures.^19−23^ Most importantly, the building blocks of luminescent MOFs, namely, metal nodes, organic ligands, and guests, can be viewed as functional modules. Note that MOF structures inherently possess slottability, allowing standardized modules to be integrated. For instance, in the synthesis of mixed-ligand MOFs, the framework imposes strict requirements on the lengths and angles of the ligands, showing higher compatibility with ligands bearing the appropriate geometries. In addition, the presence of nanosized pores allows for the ingress of guest molecules and promotes interactions between the modules and the analytes. Typically, the luminescent sensing of MOFs is achieved based on certain sensing mechanisms, as elucidated in previous studies, which mainly consist of competition absorption (CA), photoelectron transfer (PET), Förster resonance energy transfer (FRET), and structural decomposition/transformation.^24−26^ For instance, the analyte may absorb the excitation (for CA)/emission (for FRET) energy of the sensor. The energy can be transferred from the excited state of the sensor to the analyte (for PET). In some cases, the analyte may induce structural decomposition or transformation of the sensor, resulting in an immediate change in the emission intensity (Figures 1b–e).^27−31^ The diverse luminescent sensing mechanisms mentioned exhibit relative independence from one another, which, together with the structural modularity of MOFs, enables isostructural MOFs to recognize different analytes selectively. Different from conventional mixed-linker MOFs,^32,33^ this study employs two linkers with subtly varied lengths and geometries, enabling them to occupy the same positions within the framework. Obtaining single crystals of multivariate MOFs is usually challenging due to mismatches in linker length and symmetry, leading to debates about their accurate structures. Significantly, this study succeeded in attaining well-defined single-crystal structures of nine multivariate MOFs, providing direct evidence of the integration of modular linkers.

**Figure 2:**
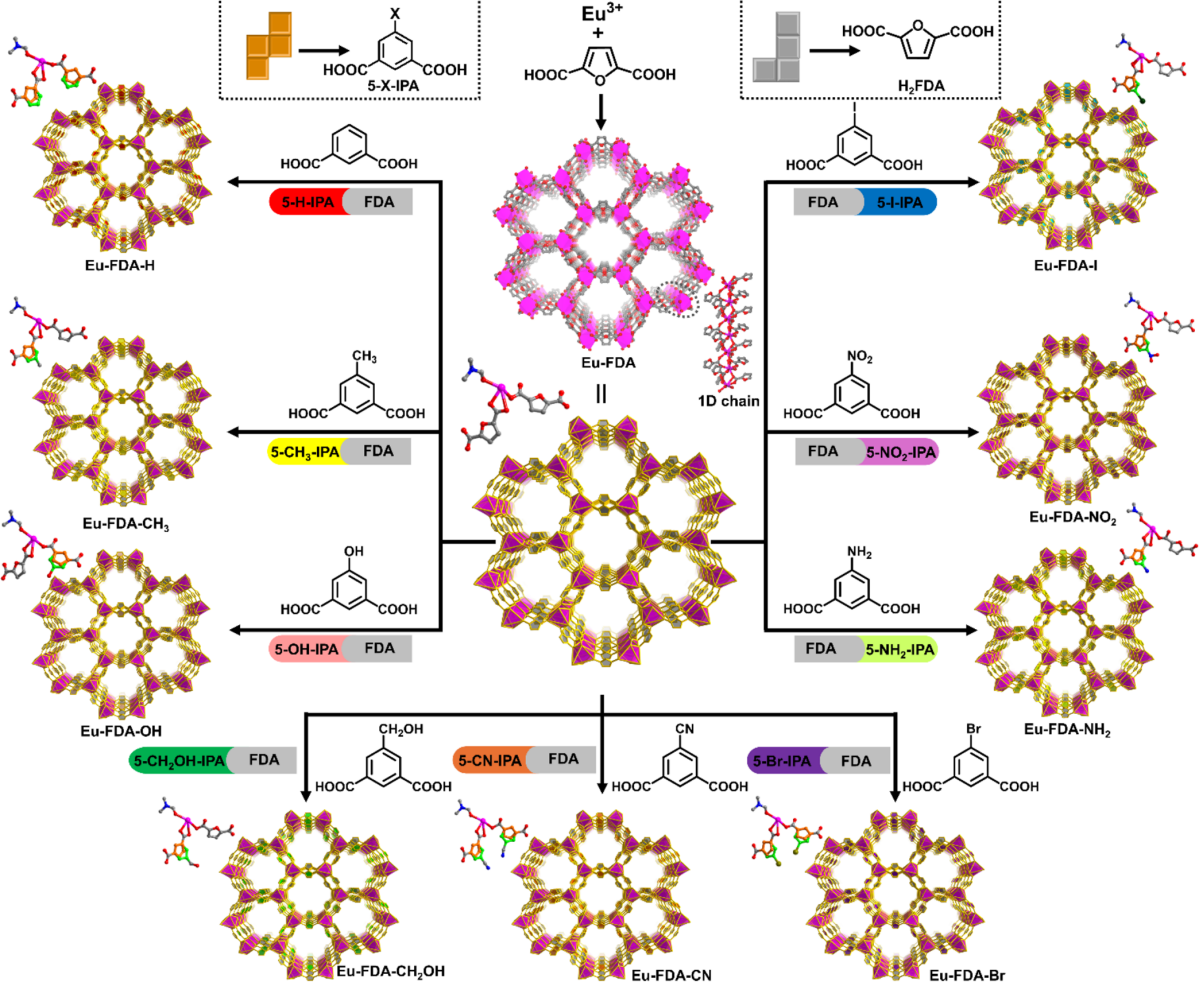
Structures of Eu-FDA series of MOFs constructed from different isophthalic acid derivatives. Atom code: C, gray; N, blue; O, red; Eu, pink; Br, dark yellow; and I, dark green. The co-occupied pentacyclic ring is colored as orange, while the co-occupied hexacyclic ring is colored as green. The rings in the topological structures have been painted as the respective background colors under the ligands.

In this work, a luminescent MOF, Eu-FDA,^34^ consisting of a one-dimensional Eu-based chain and pentacyclic 2,5-furandicarboxylic acid (H_2_FDA), was selected as the platform for the modular construction. The pentacyclic ligand of Eu-FDA can be partially replaced by a series of hexacyclic isophthalic acid derivatives, generating diverse isostructural MOFs. In a typical luminescent process, the Eu^3+^ ion serves as the luminescent center, while FDA^2–^ can function as an excellent “antenna”. The organic ligand is chosen as the variable building block, while ligands with hexacyclic rings are chosen as the alternative ligands. Compared with other building blocks and co-occupied modes, this approach allows for resolving the structures directly. Benefiting from the mixed-linker MOFs, the excellent antenna effect of FDA^2–^ and the targeting recognition function from the modular linkers can be combined to achieve a better sensing performance. The same condition applied to construct different sensors also significantly decreases the synthesis complexity and cost, compared with conventional development of sensing materials. Positions and coordination modes of the isophthalic acids are precisely defined through single-crystal X-ray diffraction (SCXRD), facilitated by the co-occupation of pentacyclic and hexacyclic ligands (Figure 2). The quantities of the loaded ligands are determined using ^1^H nuclear magnetic resonance (NMR) spectra. Presumably, when exposed to analytes with differing structures and properties, the isophthalic acid derivatives can serve as editable modules, leading to enhanced detection sensitivity based on certain sensing mechanisms.

## Results and Discussion

Pristine Eu-FDA crystals were synthesized by the reaction of Eu(NO_3_)_3_·6H_2_O, H_2_FDA, and *N*,*N*-dimethylformamide (DMF).^34^ Nine isophthalic acid derivatives, including isophthalic acid (5-H-IPA), 5-methyl-isophthalic acid (5-CH_3_-IPA), 5-bromo-isophthalic acid (5-Br-IPA), 5-iodo-isophthalic acid (5-I-IPA), 5-amino-isophthalic acid (5-NH_2_-IPA), 5-hydroxy-isophthalic acid (5-OH-IPA), 5-nitro-isophthalic acid (5-NO_2_-IPA), 5-cyano-isophthalic acid (5-CN-IPA), and 5-hydroxymethyl-isophthalic acid (5-CH_2_OH-IPA), were introduced into the framework as functional modules through one-pot synthesis with different ratios and co-occupied with FDA^2–^ (Figure 2) to generate the corresponding luminescent MOFs, namely, Eu-FDA-H, Eu-FDA-CH_3_, Eu-FDA-Br, Eu-FDA-I, Eu-FDA-NH_2_, Eu-FDA-OH, Eu-FDA-NO_2_, Eu-FDA-CN, and Eu-FDA-CH_2_OH, respectively (Table S1). These molecules were selected for their diverse structural, physical, and chemical properties. The introduced isophthalic acid derivatives induce slight changes on the unit cell parameters, while the Q peaks in the electron density map, attributed to pentacyclic rings, were changed to clearly demonstrate the co-occupation of pentacyclic and hexacyclic rings. In terms of the structure, there are minimal changes to the metal centers, carboxyl groups, and two carbon atoms directly connected to the carboxyl groups. However, the occupancies of the oxygen atom and the other two carbon atoms in FDA^2–^ decrease notably, and the benzene rings appear, presenting carboxyl groups in nearly the same positions as those in FDA^2–^.

The phase purities of all of the aforementioned MOFs were confirmed by powder X-ray diffraction (PXRD) patterns (Figure S1). ^1^H NMR spectra were used to identify the ratio between the pentacyclic and hexacyclic ligands (Figures S2–S10). Emission spectra of all of these MOFs were tested, while their various emission intensities show the different energy transfer abilities of these ligands (Figure S11). Based on the SCXRD and ^1^H NMR results, the chemical formulas of these MOFs are summarized in Table S2. Time-dependent luminescence intensities of these MOFs in DMF were recorded (Figures S12–S21), demonstrating that their luminescence intensities are stable under working conditions, which is suitable for sensing.

The sensing mechanism regarding the interactions between the modules and the analytes is the key for matching them. Eight analytes with distinct characteristics and properties were selected for analysis, including 1,2-diaminobenzene, chlorothalonil, resazurin, dodecafluorosuberic acid, perfluorooctanoic acid, nicotinamide, trimethyl borate, and hydrogen peroxide (Figure S22). To match these analytes, different isophthalic acid derivatives with certain properties were selected as the module to construct the multivariate MOFs for the sensing toward different analytes. The emission intensities originating from Eu^3+^ ions at 616 nm feature higher quenching efficiencies to target analytes in the mixed-linker MOFs compared with Eu-FDA (Figures S23–S31). The luminescence quenching efficiency at low concentrations can be quantitatively described by the linear Stern–Volmer (S–V) equation (Figures S32–S39), *I*_0_/*I* = *K*_SV_[*C*] + *b*,^35,36^ wherein *K*_SV_ is the quenching constant (M^–1^), [*C*] is the concentration of the analytes, *b* is a constant, and *I*_0_ and *I* are the luminescence intensities before and after the analyte addition, respectively. When the concentration of the analyte increased, the luminescence intensity curve gradually deviated from linear and bent upward, which can be fitted by the exponential nonlinear S–V equation, *I*_0_/*I* = *a* exp(*k*[*C*]) + *b*,^35,36^ where *a*, *b*, and *k* are constants.

**Figure 3:**
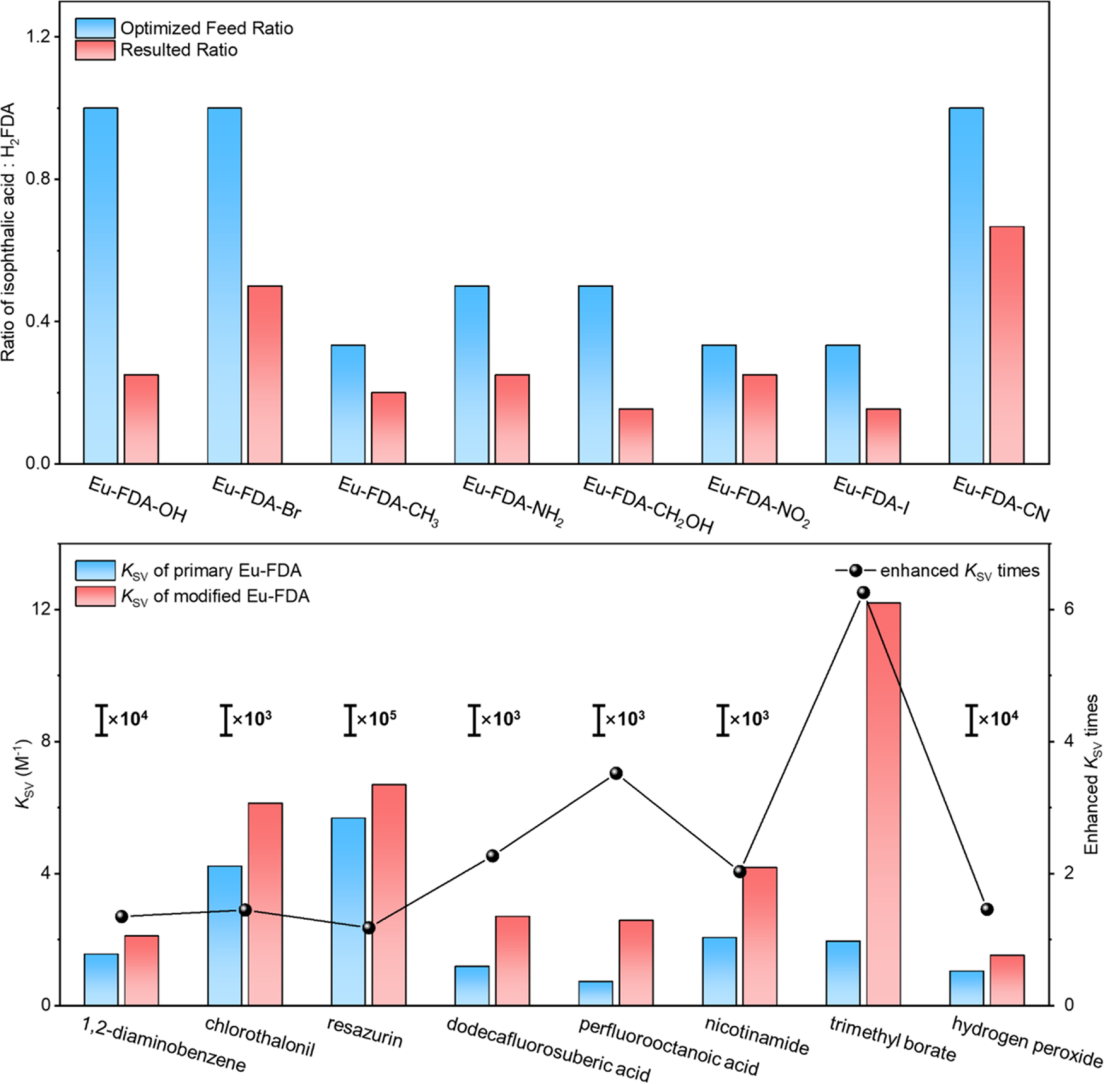
Sensing abilities of the Eu-FDA series of MOFs. Ligand ratios in the mixed-ligand Eu-MOFs and the *K*_SV_ values of these mixed-ligand Eu-MOFs toward targeting analytes compared with pristine Eu-FDA. The ligand ratios were calculated by SCXRD and ^1^H NMR spectra.

Based on the CA mechanism, 1,2-diaminobenzene was selected as the analyte, which is widely used to produce pesticide fungicides, dyes, polymer stabilizers, surfactants, and antifreeze agents. Yet, it has a strong irritant effect on the skin, which can cause severe acute eczema.^37,38^ The maximum ultraviolet (UV) absorption peak of 1,2-diaminobenzene in DMF is at around 303 nm, while the peaks of the ligand H_2_FDA and the primary MOF Eu-FDA are both at 268 nm (Figure S40). According to the CA mechanism (Figure 1b),^24^ 5-OH-IPA, featuring a more similar UV absorption peak with the analyte at around 311 nm, was selected as the module to construct the mixed-ligand MOF and Eu-FDA-OH (Figure S40), resulting in a much higher UV absorption at 303 nm and a 1.35-times higher quenching constant (Figure 3). This confirms that the regulation of the UV absorption can enhance the MOF’s sensing performance.

For the PET mechanism, chlorothalonil, a broad-spectrum fungicide that can disrupt the enzyme’s activity in fungal cells, was selected, with high adhesion to the surface of crops, which makes it difficult to be washed away. Furthermore, chlorothalonil is highly toxic to aquatic organisms, which may cause long-term adverse effects in the aquatic environment, and it has been listed as a potential carcinogen.^39,40^ As calculated, the lowest unoccupied molecular orbital (LUMO) energy level of chlorothalonil is lower than that of H_2_FDA (Figure S41), indicating that PET may occur between the ligand and the analyte (Figure 1c).^24^ Herein, 5-Br-IPA with a higher LUMO energy level was introduced into Eu-FDA, affording Eu-FDA-Br exhibiting a 1.45-times higher quenching constant (Figure 3). This confirms that the regulation of the energy level can enhance the MOF’s sensing performance.

The FRET mechanism can be selected to detect resazurin, a water-soluble nonfluorescent blue dye used as a redox indicator to evaluate viability, toxicity, proliferation, migration, and invasion in cells. As a common biological reagent, the purity of resazurin is highly valued.^41,42^ The maximum absorption peak of resazurin in DMF is measured as 638 nm, while the absorption spectrum of resazurin has obvious overlaps with the emission spectrum of Eu-FDA, allowing for a FRET process (Figure 1d).^24^ Compared to the high emission intensity of Eu-FDA, Eu-FDA-CH_3_ bearing a much lower emission intensity, which features more overlaps with the absorption of resazurin (Figure S42), was selected for the detection and obtained a 1.18-times higher quenching constant (Figure 3). This confirms that the regulation of the emission ability can enhance the MOF’s sensing performance. Further comparisons were achieved by Eu-FDA-H with a higher emission intensity than Eu-FDA and Eu-FDA-CH_3_ (Figure S42), which performs a lower quenching efficiency toward resazurin than Eu-FDA (Figures S43 and S44), confirming the importance to regulate the emission ability.

For structural decomposition/transformation, various interactions are discussed below, mainly including the structure destruction, hydrogen bond interactions, halogen bond interactions, and redox reactions. Based on the structure destruction, MOFs will be destroyed by the analytes, which can cause emission intensity changes. Long-chain perfluoroalkyl carboxylic acids were widely used as surfactants and emulsifiers. Nevertheless, nowadays they are either banned or under scrutiny because they are extremely persistent and bioaccumulative.^43,44^ Dodecafluorosuberic acid is a colorless and odorless organic compound, which is too inert to be decomposed and has cumulative toxicity in living organisms.^45^ Considering the acidity of dodecafluorosuberic acid, 5-NH_2_-IPA was introduced into the platform to lower the chemical stability of Eu-FDA to achieve the structure destruction mechanism (Figure 1e).^24^ With the addition of dodecafluorosuberic acid, the emission peak of 5-NH_2_-IPA exhibits an obvious increase (Figure S45), while the intensity changes linearly with the concentration of dodecafluorosuberic acid (Figure S46), which confirms the structure destruction of the MOF and the release of 5-NH_2_-IPA. The obtained sensor Eu-FDA-NH_2_ shows a 2.27-times higher quenching constant (Figure 3).

MOFs can serve as hydrogen bond donors in sensing. Perfluorooctanoic acid is another perfluoroalkyl carboxylic acid discovered in the 1940s and has been widely used until today, which can be found in the environment and even in the blood of animals and humans. Recently, it has been clarified as a carcinogen.^46,47^ The fluorine atoms of perfluorooctanoic acid can potentially form hydrogen bonds with MOFs. According to the literature^48^ and molecular simulation (Figure S47), hydroxyl groups can form hydrogen bonding with fluorine atoms. Herein, 5-CH_2_OH-IPA was selected as the hydrogen bond donor in the framework, which shows a 3.52-times higher quenching constant (Figure 3).

In addition, MOFs can also serve as hydrogen bond acceptors in sensing. Nicotinamide is found in food and used as a dietary supplement and medication, which is the preferred treatment for pellagra caused by niacin deficiency with limited side effects.^49,50^ According to the literatures^51−53^ and molecular simulation (Figure S48), nitro groups can form hydrogen bonds with amide groups. Herein, Eu-FDA-NO_2_ was synthesized to enhance the sensing properties, wherein 5-NO_2_-IPA served as the hydrogen bond acceptor, affording a 2.03-times higher quenching constant (Figure 3).

Similarly, other weak interactions such as halogen bonds can be utilized for sensing. Trimethyl borate is an industrial raw material, which is mainly used as a vulcanizing agent, wood preservative, catalyst, gelling agent, heat stabilizer, and flame-extinguishing agent and for the preparation of active silica.^54,55^ According to the literatures^56,57^ and molecular simulation (Figure S49), the oxygen atoms in trimethyl borate can form halogen bonds with halogen atoms, especially iodine atoms. Therefore, 5-I-IPA can serve as the potential binding site to accommodate trimethyl borate, while the modified sensor exhibits a 6.26-times higher quenching constant (Figure 3).

Moreover, redox reactions can also function as driving forces for sensing. Hydrogen peroxide is widely used as an oxidant, bleach, disinfectant, dechlorinating agents, rocket fuel, and foam plastic.^58,59^ In literature, hydrogen peroxide can oxidize cyanide groups under mild conditions.^60^ An obvious shift can be found in the ^13^C NMR, UV, and mass spectra of 5-CN-IPA before and after the hydrogen peroxide addition, indicating the occurrence of the redox reaction (Figures S50–S52). In this case, 5-CN-IPA was inserted into the MOF, resulting in a 1.46-times higher quenching constant (Figure 3).

**Figure 4:**
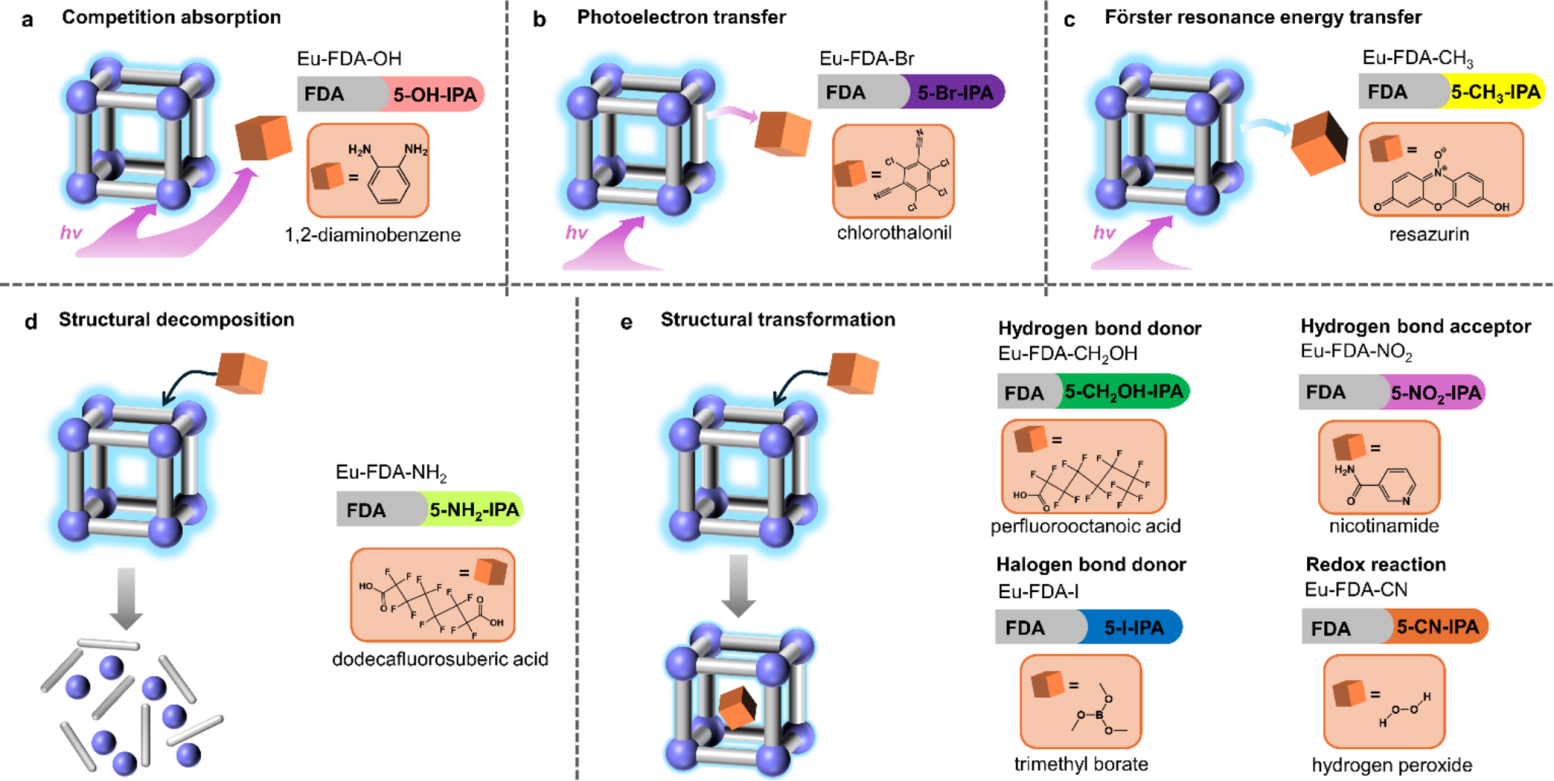
Schematic illustration of sensing mechanisms of the Eu-FDA-derived MOFs. (a) Competition absorption between Eu-FDA-OH and 1,2-diaminobenzene. (b) Photoelectron transfer from Eu-FDA-Br to chlorothalonil. (c) Förster resonance energy transfer from Eu-FDA-CH_3_ to resazurin. (d) Structural decomposition of Eu-FDA-NH_2_ by dodecafluorosuberic acid. (e) Structural transformation of Eu-FDA-CH_2_OH by perfluorooctanoic acid through a hydogen bond, Eu-FDA-NO_2_ by nicotinamide through a hydogen bond, Eu-FDA-I by trimethyl borate through a halogen bond, and Eu-FDA-CN by hydrogen peroxide through a redox reaction.

On the basis of the above design and discussion, the Eu-based MOF was prepared to provide a versatile platform for luminescent sensing. Different mixed ligands can be introduced into the platform as functional modules, affording MOFs with the desired spectra, energy level, and functional groups. The resultant MOFs feature a largely enhanced sensing capability toward eight distinct analytes, based on various sensing mechanisms including CA, PET, FRET, and structural decomposition/transformation (Figure 4). In summary, good luminescence properties stem from the antenna effect of the FDA linker, which forms the foundation of an effective luminescent sensor. When targeting analytes with specific properties, the modular linkers 5-X-IPA play a more crucial role due to their superior compatibility. This work presents one intricately designed case of how the modularity of MOFs can be utilized for highly efficient and selective luminescent sensing in an unprecedented facile manner.

## Conclusions

In this work, a Eu-MOF-based luminescent sensing platform was established, while pentacyclic and hexacyclic ring mixed ligands were employed as modules for detecting analytes with various structural and property characteristics. The pentacyclic 2,5-furandicarboxylic acid and hexacyclic isophthalic acid derivatives can occupy the same position, confirmed through SCXRD and ^1^H NMR spectra. Analytes with certain absorption spectra, energy levels, and functional groups can be specifically sensed by this slot-type platform through introducing specific modules, based on distinct sensing mechanisms, including CA, PET, FRET, and structural decomposition/transformation. From a synthetic perspective, this work has developed a novel modular MOF Eu-FDA platform, which, through facile ligand replacement, can be derived into multivariate MOFs for the selective recognition of eight substrates with different structures and properties, confirming its robustness and versatility, which is also comparable with literatures (Table S3). Leveraging the modularity of MOFs and the slottability of organic ligands, we present the first example of modular design in luminescent sensing materials, enabling precise synthesis tailored according to the structures and properties of analytes. It is expected that this modular design strategy will overcome the limitations of narrow substrate scopes and low specificity in luminescent recognition, ultimately facilitating the advancement of novel materials with application potential in precision medicine and environmental protection.

## Supporting Information Available

Experimental details, basic characterizations of the relative MOFs, luminescent sensing, sensing mechanism, and relative tables for these MOFs toward analytes (PDF)
