# Supplementary material for: Modular Construction of Multivariate Metal–Organic Frameworks for Luminescent Sensing
Source: J Am Chem Soc. 2025 Jan 15;147(4):3866–73. doi: 10.1021/jacs.4c17248 (PMC11783584; doi:10.1021/jacs.4c17248)
Supplement: Supplementary file 1 — ja4c17248_si_001.pdf [file ja4c17248_si_001.pdf]

Supporting Information

Modular Construction of Multivariate Metal-Organic Frameworks for Luminescent Sensing

Zongsu Han,[†,§] Kun-Yu Wang,[†,§] Rong-Ran Liang,[†,§] Yifan Guo,[‡] Yihao Yang,[†] Mengmeng Wang,[⊥] Yue Mao,[⊥] Jiatong Huo,[†] Wei Shi,[⊥,*] and Hong-Cai Zhou[†,*]

[†]Department of Chemistry, Texas A&M University, College Station, Texas 77843, United States

[‡]Health Science Platform, Tianjin University, A203, Bldg. 24, 92 Weijin Rd., Nankai Dist., Tianjin 300072, China

[⊥]Frontiers Science Center for New Organic Matter, Key Laboratory of Advanced Energy Materials Chemistry (MOE), and State Key Laboratory of Advanced Chemical Power Sources, College of Chemistry, Nankai University, Tianjin 300071, China

Contents

Experimental	2
Basic characterizations	6
Luminescence sensing	17
Sensing mechanisms	27
Tables	34
References	38

Experimental

Materials.

All reagents were commercially available and used without further purification. Liquid NMR spectra were recorded on a Bruker Avance NEO 400 NMR spectrometer. SCXRD patterns were collected by a Rigaku XtaLAB Synergy-S diffractometer with Mo/Cu-$K\alpha$ radiation. The structures were solved by SHELXS (direct methods) and refined by SHELXL (full matrix least-squares techniques) in the Olex2 package.[1,2] PXRD measurements were performed using a Bruker Powder-ECO X-ray diffractometer with Cu-Kα radiation. Luminescence spectra were recorded on a Shimadzu RF-5301 fluorescence spectrophotometer. UV-vis absorption spectra were measured by a Shimadzu UV-2450 Spectrometer. Mass spectra are collected by a Thermo Scientific Qexactive Focus equipment. For NMR tests, 3 mg ligands were dissolved by 0.5 mL d_6-DMSO, while 3 mg MOFs were dissolved by 0.5 mL d_6-DMSO and 5 μL D_2SO_4. Quantum mechanical calculation program Turbomole 7.5 was implemented. Def-TZVP/m06-2x was used for geometry optimizations and calculating the single-point energy.

Methods.

Synthesis of Eu-FDA

Eu-FDA was synthesized referring to the literature.[3] A mixture of $Eu(NO_3)_3 \cdot 6\,H_2O$ (44.6 mg, 0.1 mmol), H_2FDA (31.2 mg, 0.2 mmol), and DMF 2 mL was added in a 20 mL sealed glass bottle under ultrasound, and then heated at 120 °C for one day. Then the crystals were collected by filtration, washed with fresh DMF, and soaked in fresh DMF for night.

Different ratios of the hybrid ligands were selected relying on the crystallinities of the samples. Higher ratios of the isophthalic acid derivatives than selected may lead to no suitable single crystals.

Synthesis of Eu-FDA-H

A mixture of $Eu(NO_3)_3 \cdot 6\,H_2O$ (44.6 mg, 0.1 mmol), H_2FDA (7.8 mg, 0.05 mmol), 5-H-IPA (24.9 mg, 0.15 mmol), and DMF 2 mL was added in a 20 mL sealed glass bottle under ultrasound, and then heated at 120 °C for one day. Then the crystals were collected by filtration, washed with fresh DMF, and soaked in fresh DMF for night.

Synthesis of Eu-FDA-CH_3

A mixture of $Eu(NO_3)_3 \cdot 6\,H_2O$ (44.6 mg, 0.1 mmol), H_2FDA (23.4 mg, 0.15 mmol), 5-CH_3-IPA (9.0 mg, 0.05 mmol), and DMF 2 mL was added in a 20 mL sealed glass bottle under ultrasound, and then heated at 120 °C for one day. Then the crystals were collected by filtration, washed with fresh DMF, and soaked in fresh DMF for night.

Synthesis of Eu-FDA-Br

A mixture of $Eu(NO_3)_3 \cdot 6\,H_2O$ (44.6 mg, 0.1 mmol), H_2FDA (15.6 mg, 0.1 mmol), 5-Br-IPA (24.5 mg, 0.1 mmol), and DMF 2 mL was added in a 20 mL sealed glass bottle under ultrasound, and then heated at 120 °C for one day. Then the crystals were collected by filtration, washed with fresh DMF, and soaked in fresh DMF for night.

Synthesis of Eu-FDA-I

A mixture of $Eu(NO_3)_3 \cdot 6\,H_2O$ (44.6 mg, 0.1 mmol), H_2FDA (23.4 mg, 0.15 mmol), 5-I-IPA (14.6 mg, 0.05 mmol), and DMF 2 mL was added in a 20 mL sealed glass bottle under ultrasound, and then heated at 120 °C for one day. Then the crystals were collected by filtration, washed with fresh DMF, and soaked in fresh DMF for night.

Synthesis of Eu-FDA-NH_2

A mixture of $Eu(NO_3)_3 \cdot 6\,H_2O$ (44.6 mg, 0.1 mmol), H_2FDA (20.8 mg, 0.13 mmol), 5-NH_2-IPA (12.1 mg, 0.07 mmol), and DMF 2 mL was added in a 20 mL sealed glass bottle under ultrasound, and then heated at 120 °C for one day. Then the crystals were collected by filtration, washed with fresh DMF, and soaked in fresh DMF for night.

Synthesis of Eu-FDA-OH

A mixture of $Eu(NO_3)_3 \cdot 6\,H_2O$ (44.6 mg, 0.1 mmol), H_2FDA (15.6 mg, 0.1 mmol), 5-OH-IPA (18.2 mg, 0.1 mmol), and DMF 2 mL was added in a 20 mL sealed glass bottle under ultrasound, and then heated at 120 °C for one day. Then the crystals were collected by filtration, washed with fresh DMF, and soaked in fresh DMF for night.

Synthesis of Eu-FDA-NO_2

A mixture of $Eu(NO_3)_3 \cdot 6\,H_2O$ (44.6 mg, 0.1 mmol), H_2FDA (23.4 mg, 0.15 mmol), 5-NO_2-IPA (10.6 mg, 0.05 mmol), and DMF 2 mL was added in a 20 mL sealed glass bottle under ultrasound, and then heated at 120 °C for one day. Then the crystals were collected by filtration, washed with fresh DMF, and soaked in fresh DMF for night.

Synthesis of Eu-FDA-CN

A mixture of $Eu(NO_3)_3 \cdot 6\,H_2O$ (44.6 mg, 0.1 mmol), H_2FDA (15.6 mg, 0.1 mmol), 5-CN-IPA (19.1 mg, 0.1 mmol), and DMF 2 mL was added in a 20 mL sealed glass bottle under ultrasound, and then heated at 120 °C for one day. Then the crystals were collected by filtration, washed with fresh DMF, and soaked in fresh DMF for night.

Synthesis of Eu-FDA-CH_2OH

A mixture of $Eu(NO_3)_3 \cdot 6\,H_2O$ (44.6 mg, 0.1 mmol), H_2FDA (20.8 mg, 0.13 mmol), 5-CH_2OH-IPA (13.1 mg, 0.07 mmol), and DMF 2 mL was added in a 20 mL sealed glass bottle under ultrasound, and then heated at 120 °C for one day. Then the crystals were collected by filtration, washed with fresh DMF, and soaked in fresh DMF for night.

Luminescence sensing experiments

All samples were grinded into fine powder before use. The samples for luminescence experiments were dispersed in DMF by ultrasound for 10 minutes to form a clear suspension with a concentration of 1.0 mg mL^{-1}. Analytes were diluted by DMF, which were further added to the MOF suspension. Each line was tested after 10 seconds ultrasound. All the emission spectra were excited under 287 nm.

Energy level calculations

Portions of this research were conducted with high performance research computing resources provided by Texas A&M University (https://hprc.tamu.edu). All DFT calculations were performed using ORCA software package (version 5.0.4).[4,5] The geometry optimization for all molecules were carried out at r^2SCAN-3c/def2-mTZVVP/def2-mTZVVP/J[6] level of theory. All calculations were corrected utilizing the geometrical counterpoise correction gCP[7] and the atom-pairwise dispersion correction based on tight binding partial charges (D4).[8,9] Frequency calculations were performed to validate each structure at minimum (no imaginary frequencies). The free energies for all structures were calculated at 298.15 K and 1.0 atm using the same level as geometry optimization.

Basic characterizations

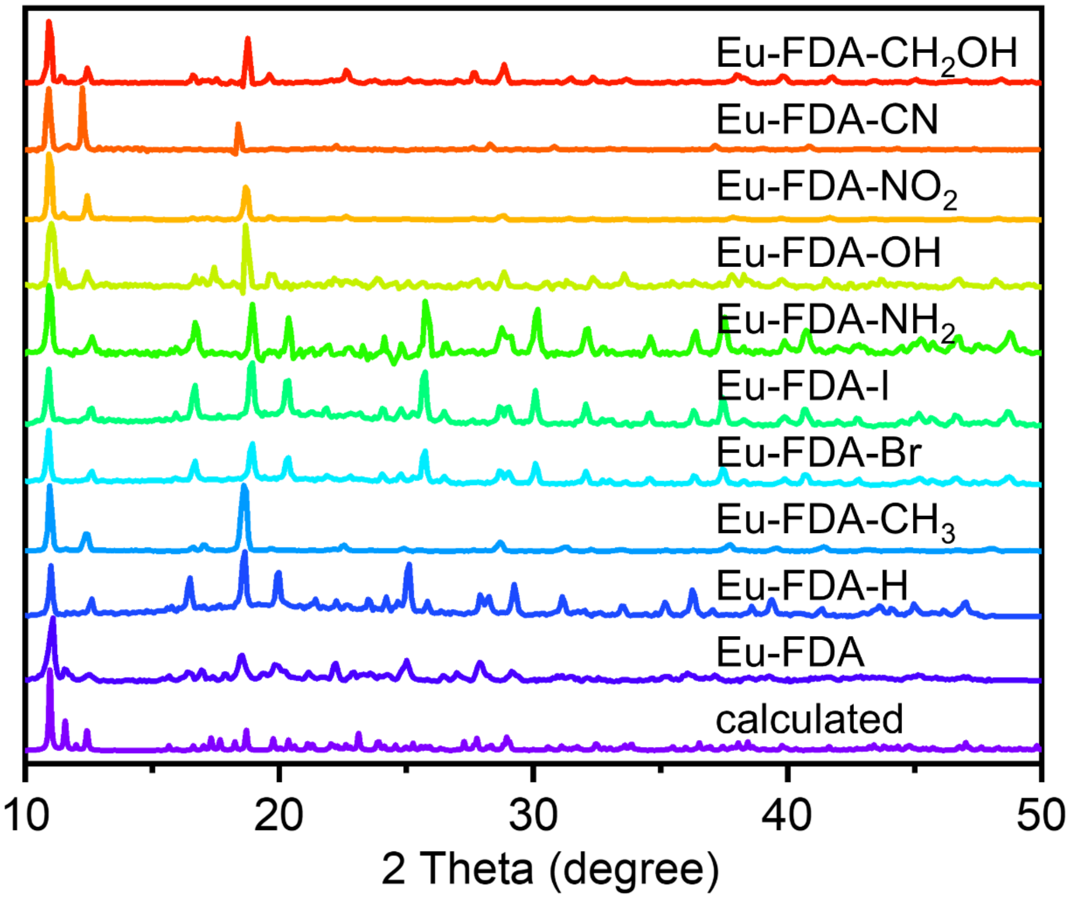

Figure S1. PXRD patterns of calculated and synthesized Eu-FDA series MOFs.

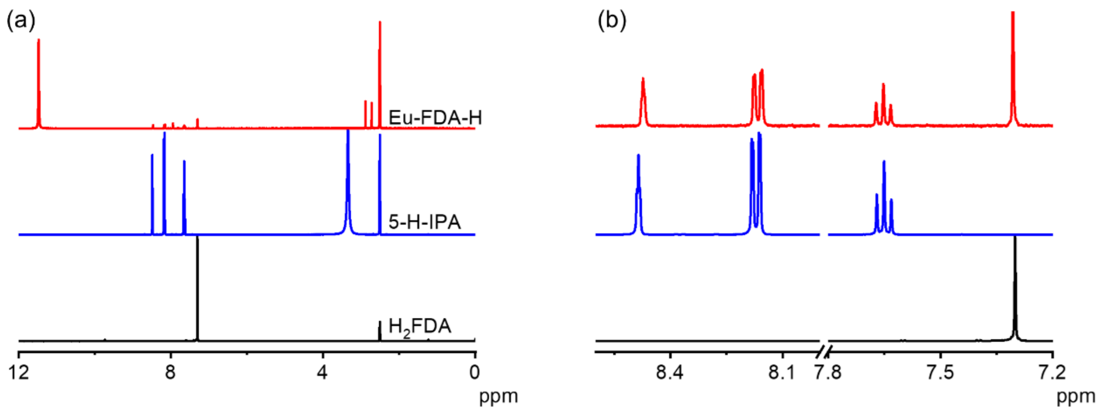

Figure S2. Liquid ^{1}H NMR spectra and partial enlarged figure of H_2FDA, 5-H-IPA, and Eu-FDA-H.

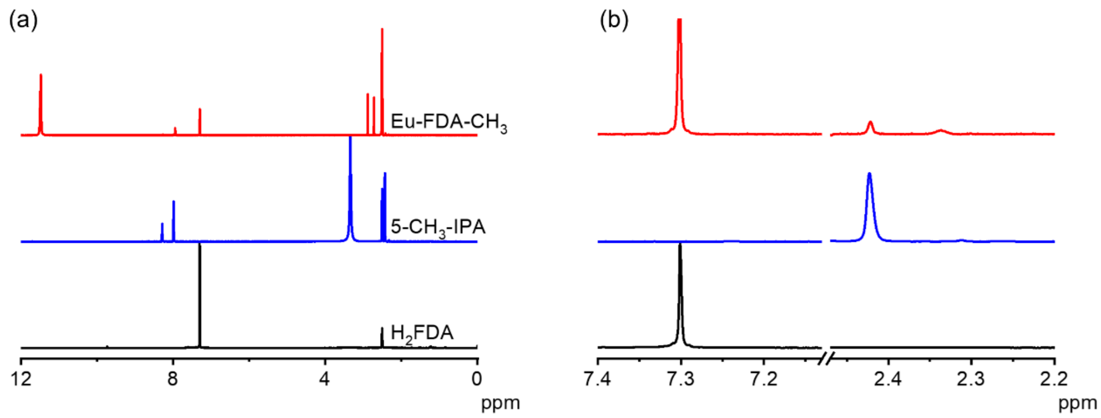

Figure S3. Liquid ^{1}H NMR spectra and partial enlarged figure of H_2FDA, 5-CH_3-IPA, and Eu-FDA-CH_3.

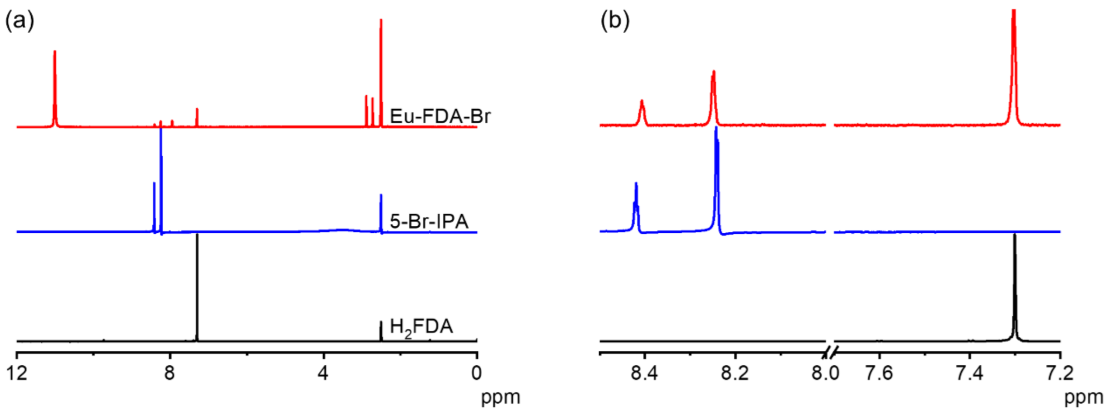

Figure S4. Liquid ^{1}H NMR spectra and partial enlarged figure of H_2FDA, 5-Br-IPA, and Eu-FDA-Br.

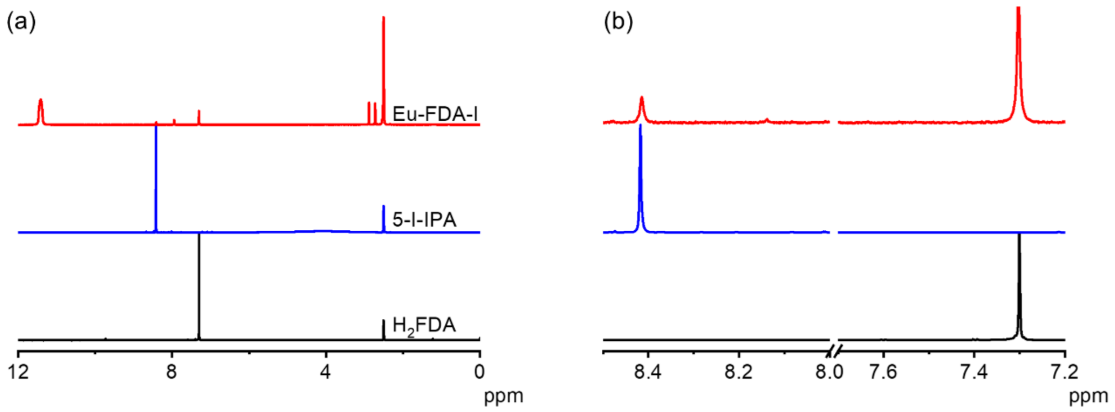

Figure S5. Liquid ^{1}H NMR spectra and partial enlarged figure of H_2FDA, 5-I-IPA, and Eu-FDA-I.

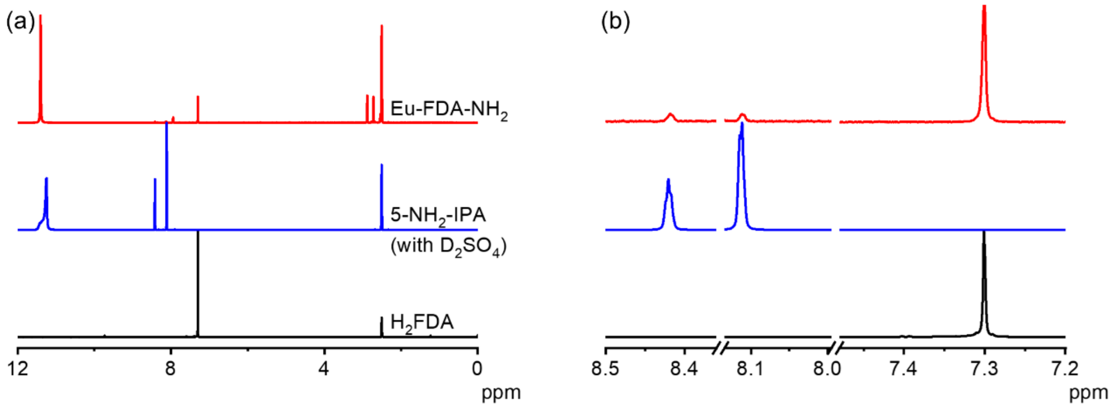

Figure S6. Liquid ^{1}H NMR spectra and partial enlarged figure of H_2FDA, 5-NH_2-IPA (with D_2SO_4), and Eu-FDA-NH_2. Due to the obviously different chemical shifts of 5-NH_2-IPA without and with D_2SO_4, this figure exhibits the NMR spectrum of 5-NH_2-IPA with 5 μL D_2SO_4.

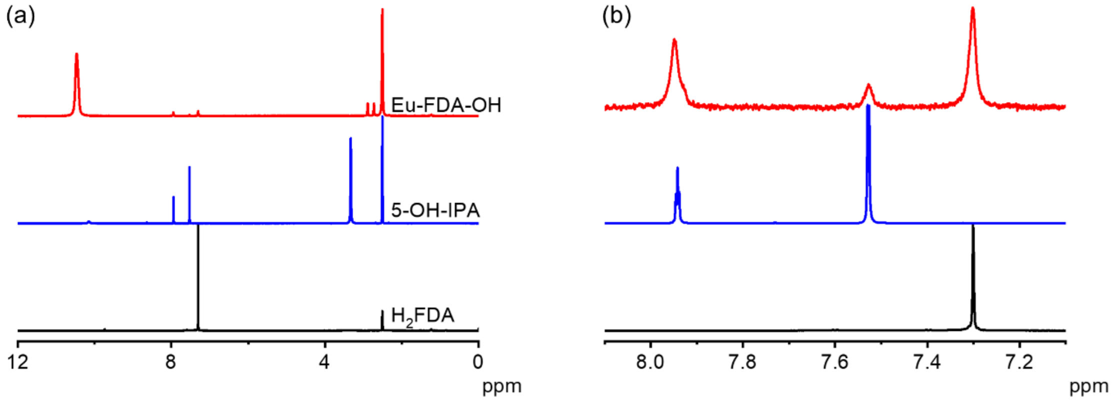

Figure S7. Liquid ^{1}H NMR spectra and partial enlarged figure of H_2FDA, 5-OH-IPA, and Eu-FDA-OH.

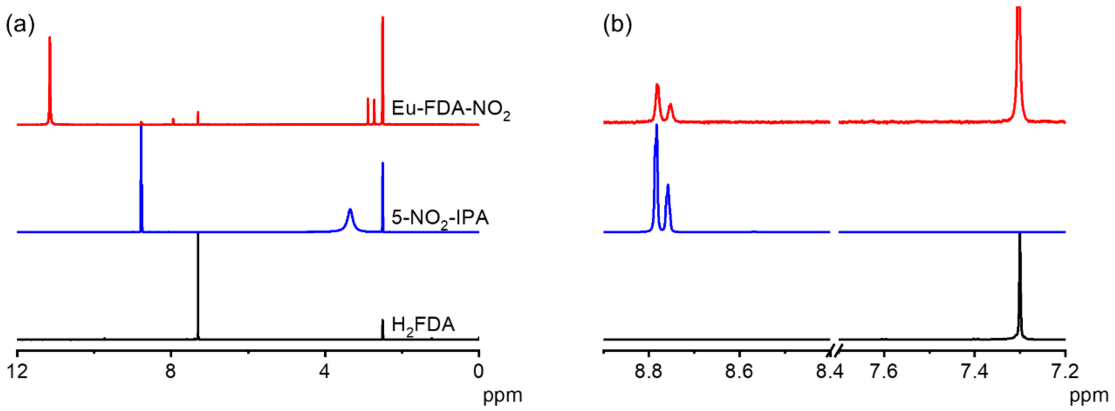

Figure S8. Liquid ^{1}H NMR spectra and partial enlarged figure of H_2FDA, 5-NO_2-IPA, and Eu-FDA-NO_2.

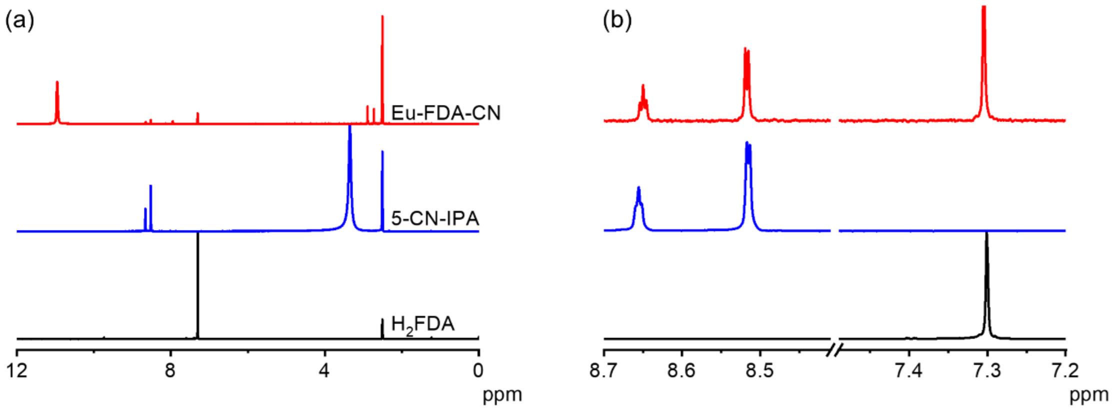

Figure S9. Liquid ^{1}H NMR spectra and partial enlarged figure of H_2FDA, 5-CN-IPA, and Eu-FDA-CN.

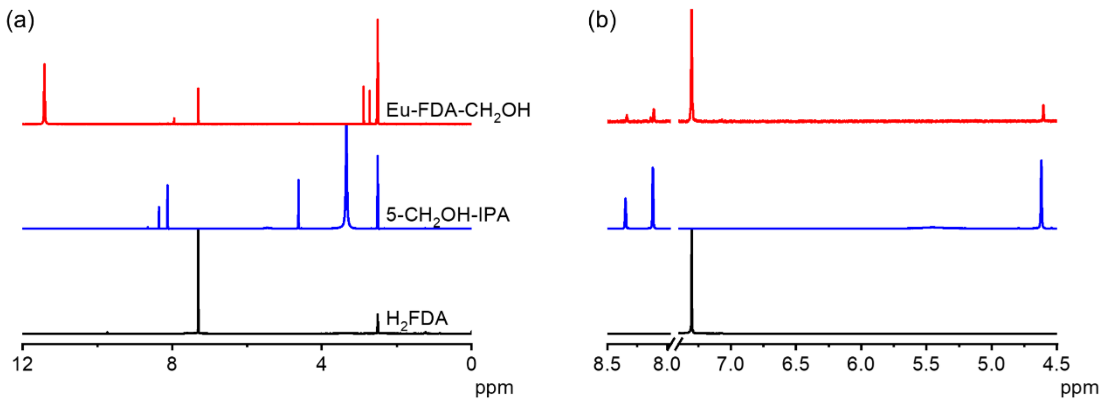

Figure S10. Liquid ^{1}H NMR spectra and partial enlarged figure of H_2FDA, 5-CH_2OH-IPA, and Eu-FDA-CH_2OH.

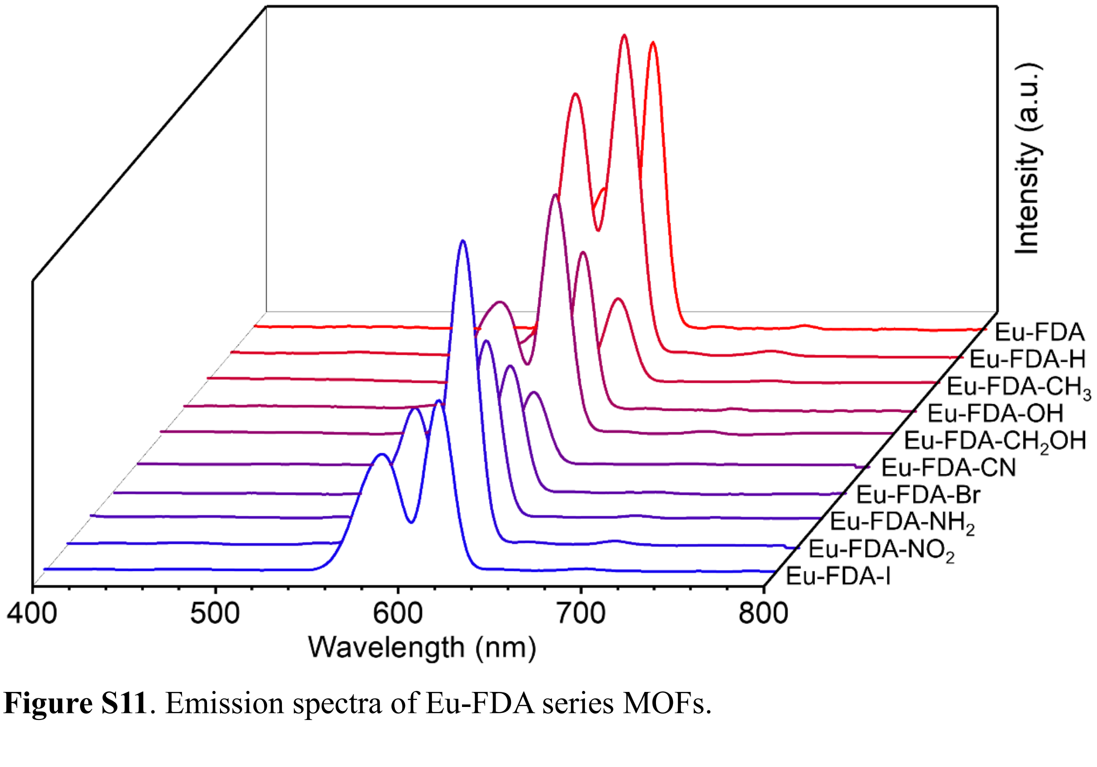

Figure S11. Emission spectra of Eu-FDA series MOFs.

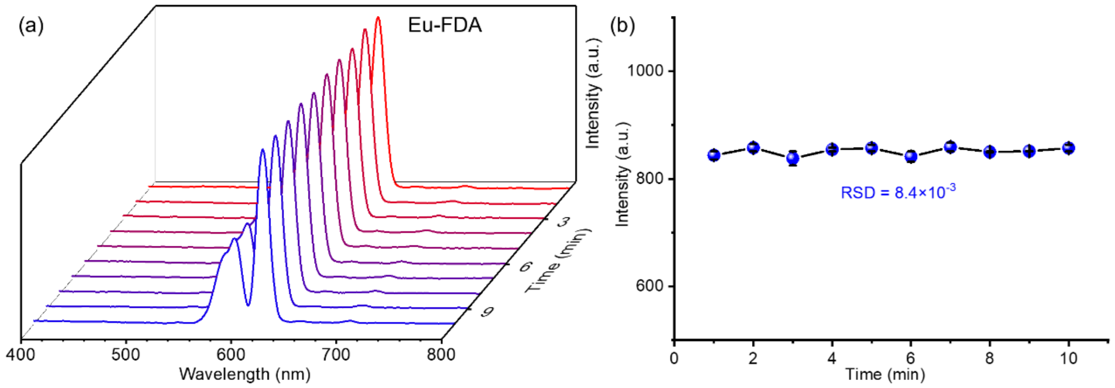

Figure S12. Time-dependent emission spectra (a) and luminescence intensities (b) of Eu-FDA in DMF.

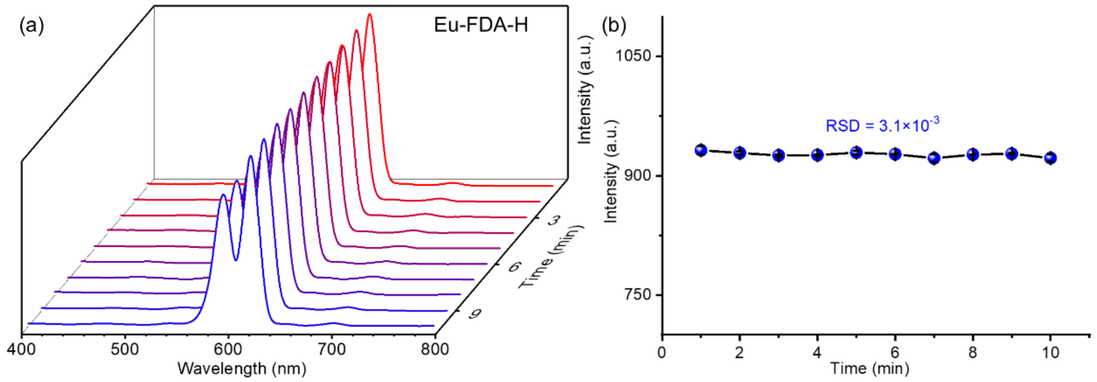

Figure S13. Time-dependent emission spectra (a) and luminescence intensities (b) of Eu-FDA-H in DMF.

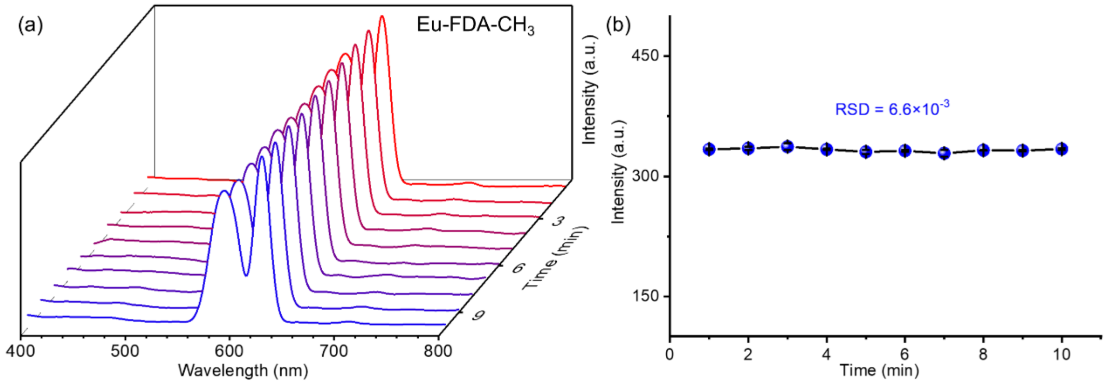

Figure S14. Time-dependent emission spectra (a) and luminescence intensities (b) of Eu-FDA-CH_3 in DMF.

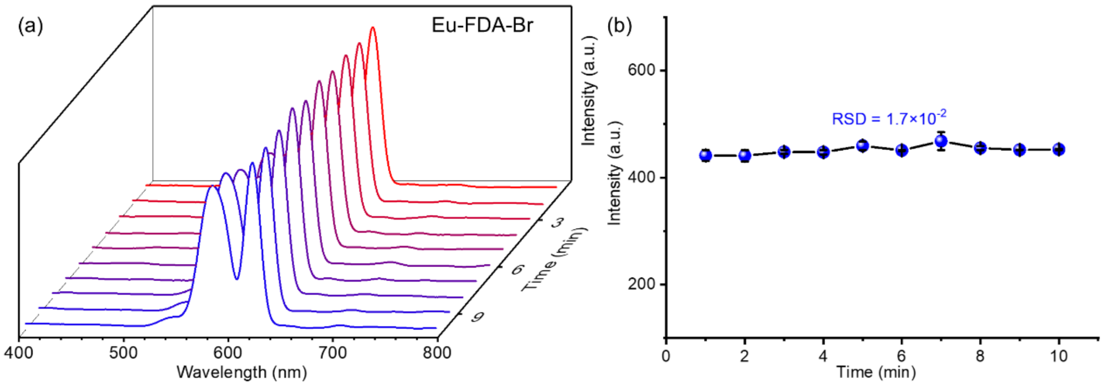

Figure S15. Time-dependent emission spectra (a) and luminescence intensities (b) of Eu-FDA-Br in DMF.

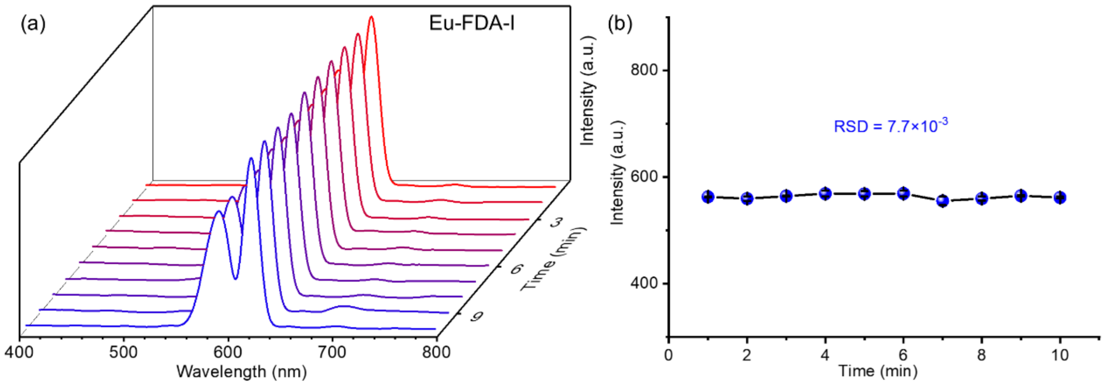

Figure S16. Time-dependent emission spectra (a) and luminescence intensities (b) of Eu-FDA-I in DMF.

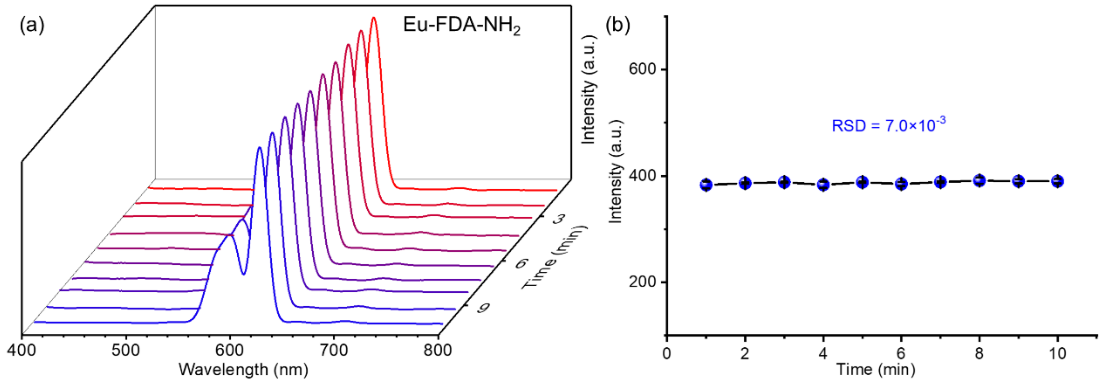

Figure S17. Time-dependent emission spectra (a) and luminescence intensities (b) of Eu-FDA-NH_2 in DMF.

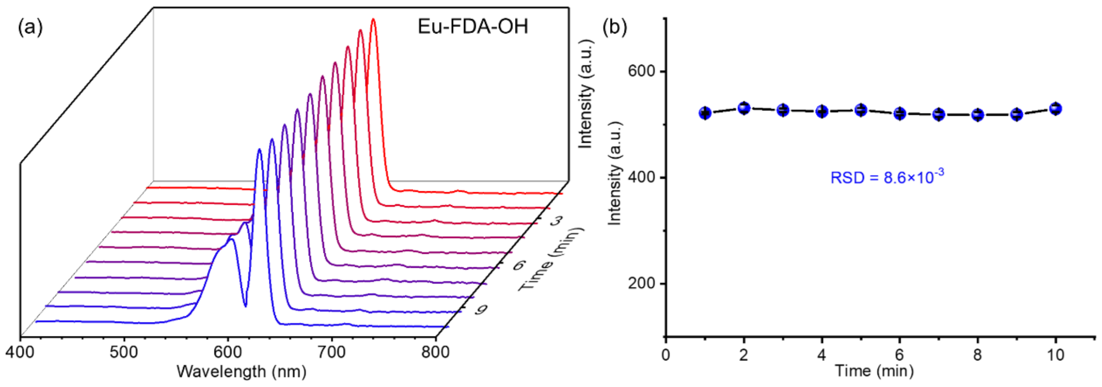

Figure S18. Time-dependent emission spectra (a) and luminescence intensities (b) of Eu-FDA-OH in DMF.

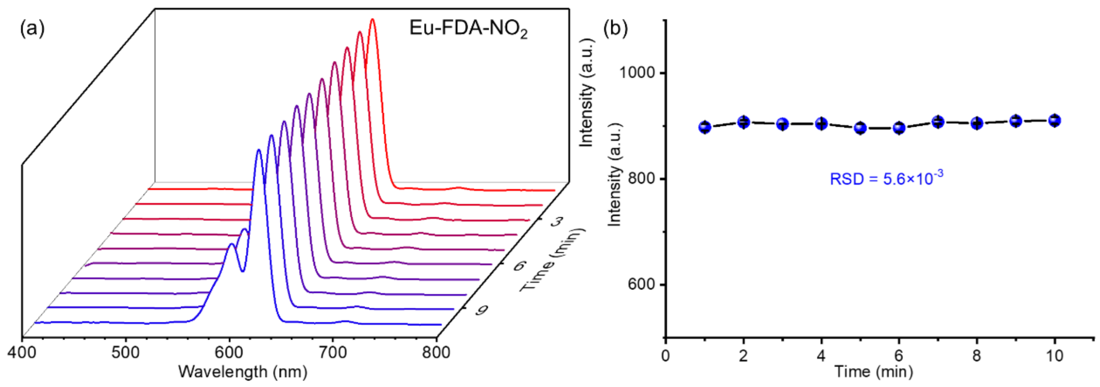

Figure S19. Time-dependent emission spectra (a) and luminescence intensities (b) of Eu-FDA-NO_2 in DMF.

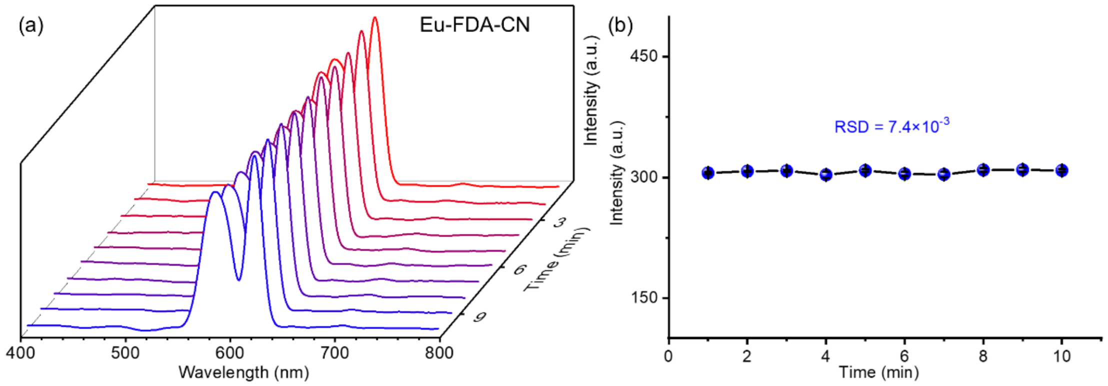

Figure S20. Time-dependent emission spectra (a) and luminescence intensities (b) of Eu-FDA-CN in DMF.

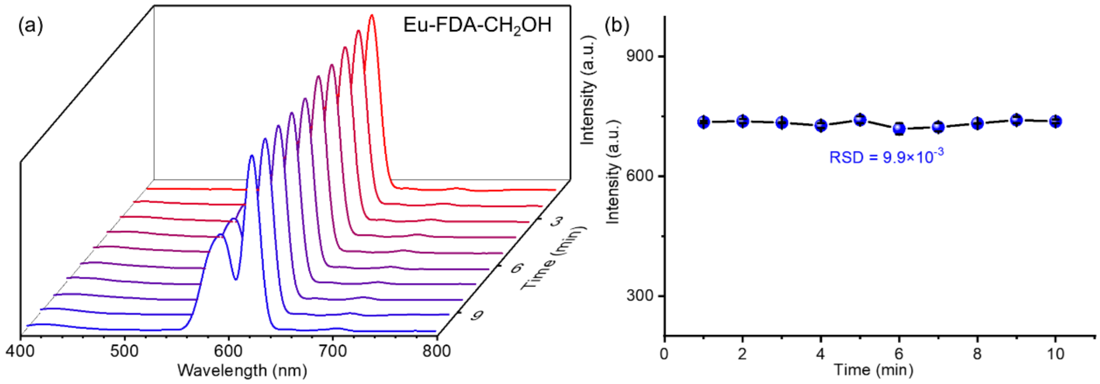

Figure S21. Time-dependent emission spectra (a) and luminescence intensities (b) of Eu-FDA-CH_2OH in DMF.

Luminescence sensing

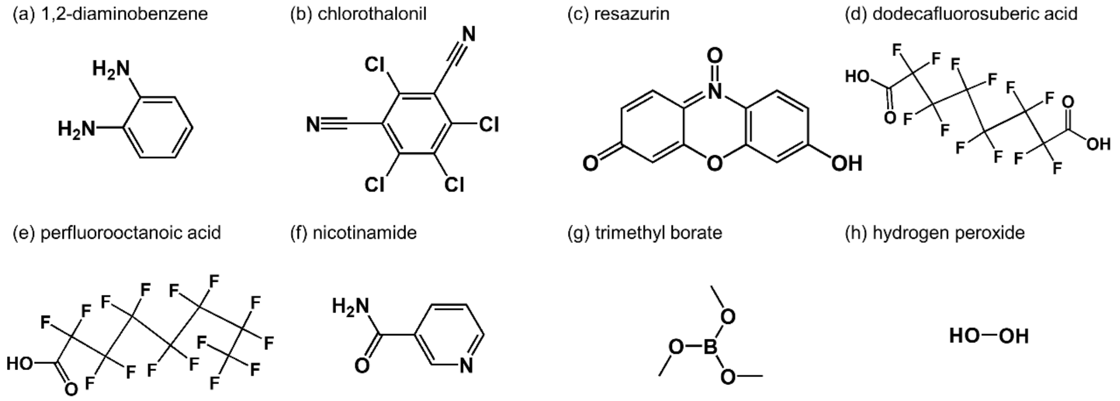

Figure S22. Structures of the analytes.

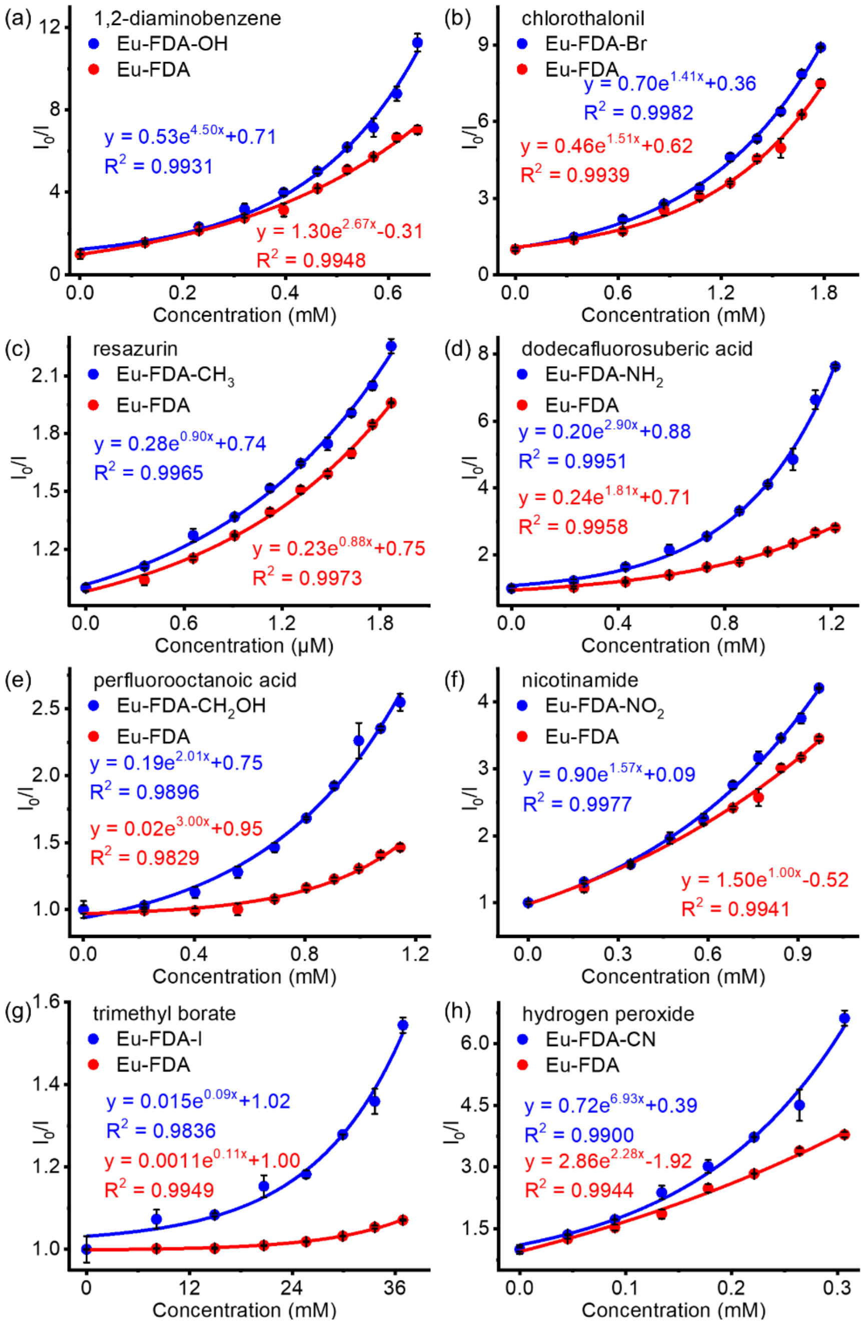

Figure S23. Emission intensity changes of (a) Eu-FDA-OH and Eu-FDA towards additions of 1,2-diaminobenzene; (b) Eu-FDA-Br and Eu-FDA towards additions of chlorothalonil; (c) Eu-FDA-CH_3 and Eu-FDA towards additions of resazurin; (d) Eu-FDA-NH_2 and Eu-FDA towards additions of dodecafluorosuberic acid; (e) Eu-FDA-CH_2OH and Eu-FDA towards additions of perfluorooctanoic acid; (f) Eu-FDA-NO_2 and Eu-FDA towards additions of nicotinamide; (g) Eu-FDA-I and Eu-FDA towards additions of trimethyl borate; (h) Eu-FDA-CN and Eu-FDA towards additions of hydrogen peroxide.

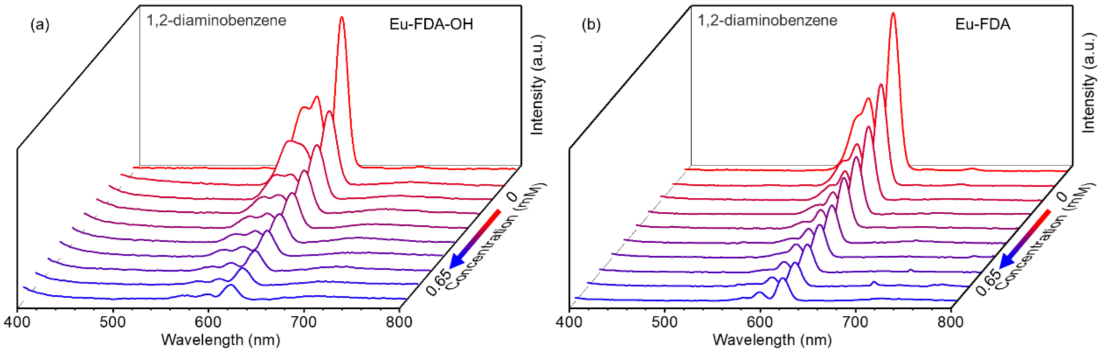

Figure S24. Emission spectra of Eu-FDA-OH (a) and Eu-FDA (b) towards additions of 1,2-diaminobenzene.

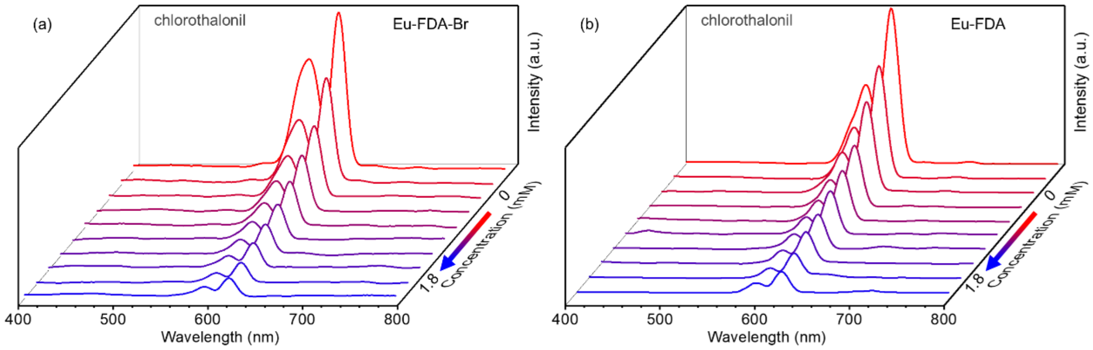

Figure S25. Emission spectra of Eu-FDA-Br (a) and Eu-FDA (b) towards additions of chlorothalonil.

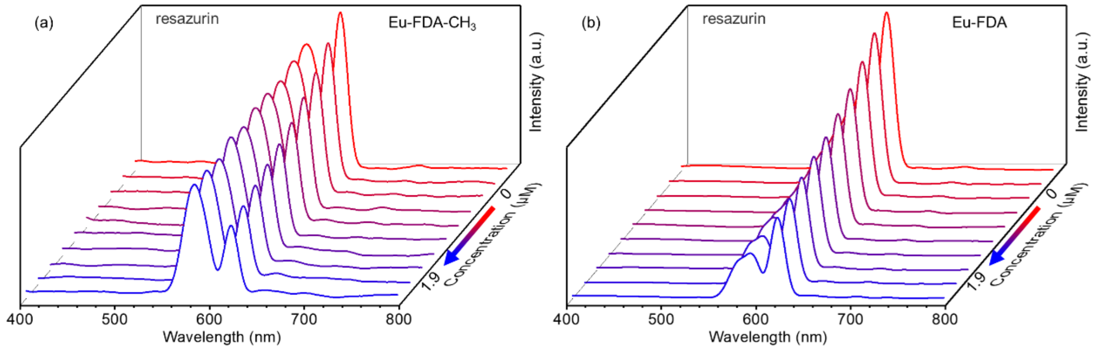

Figure S26. Emission spectra of Eu-FDA-CH_3 (a) and Eu-FDA (b) towards additions of resazurin.

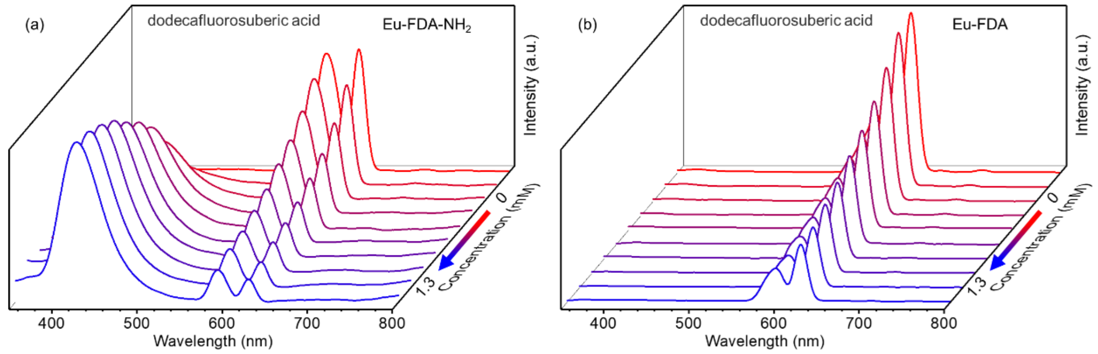

Figure S27. Emission spectra of Eu-FDA-NH_2 (a) and Eu-FDA (b) towards additions of dodecafluorosuberic acid.

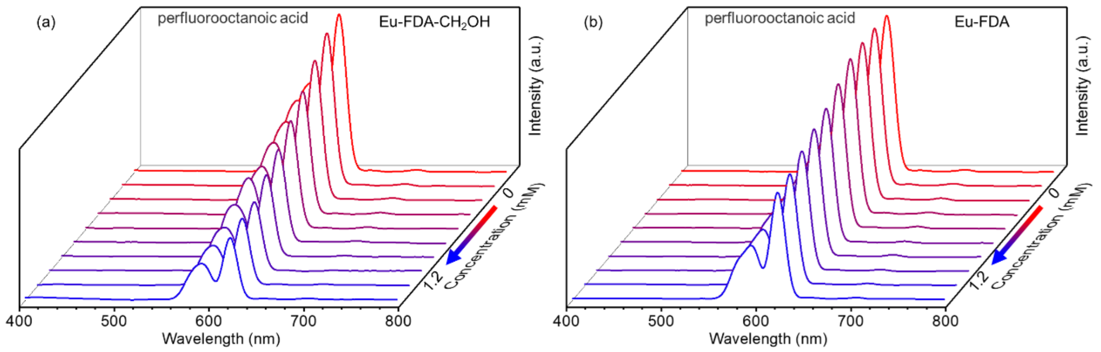

Figure S28. Emission spectra of Eu-FDA-CH_2OH (a) and Eu-FDA (b) towards additions of perfluorooctanoic acid.

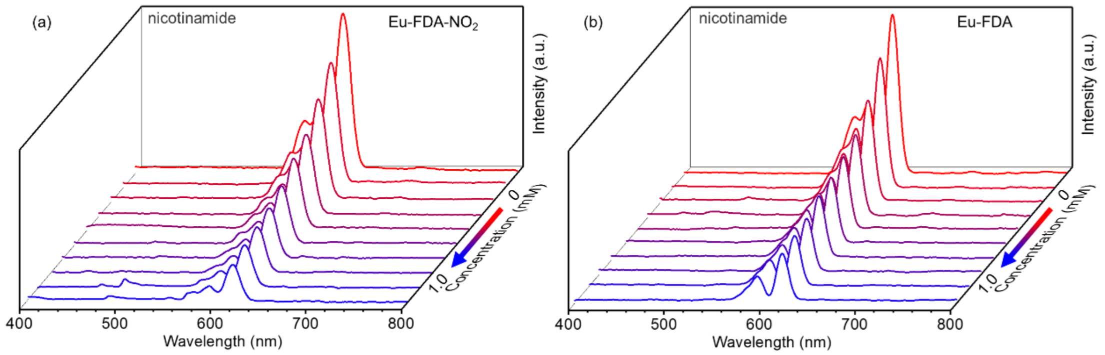

Figure S29. Emission spectra of Eu-FDA-NO_2 (a) and Eu-FDA (b) towards additions of nicotinamide.

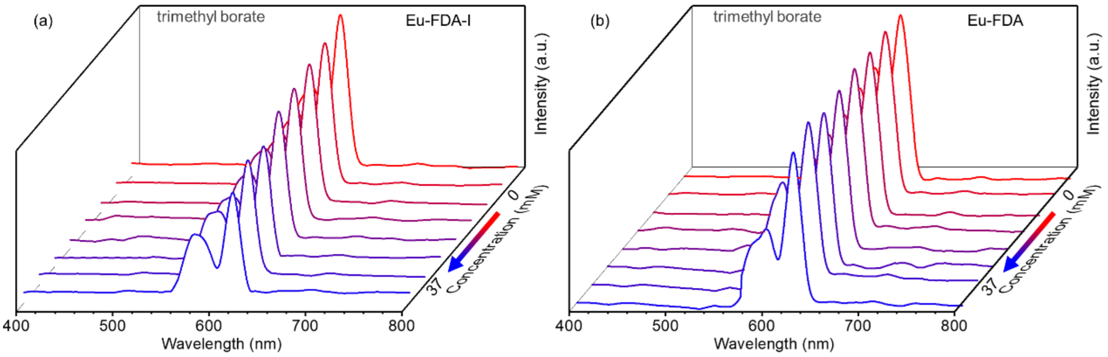

Figure S30. Emission spectra of Eu-FDA-I (a) and Eu-FDA (b) towards additions of trimethyl borate.

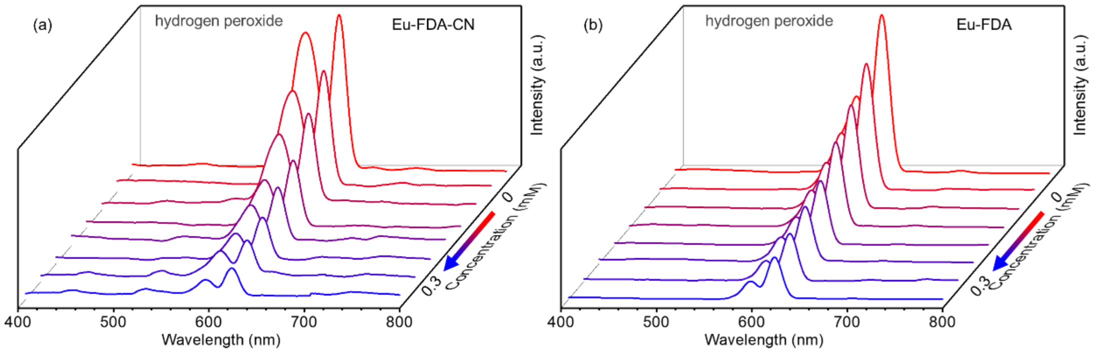

Figure S31. Emission spectra of Eu-FDA-CN (a) and Eu-FDA (b) towards additions of hydrogen peroxide.

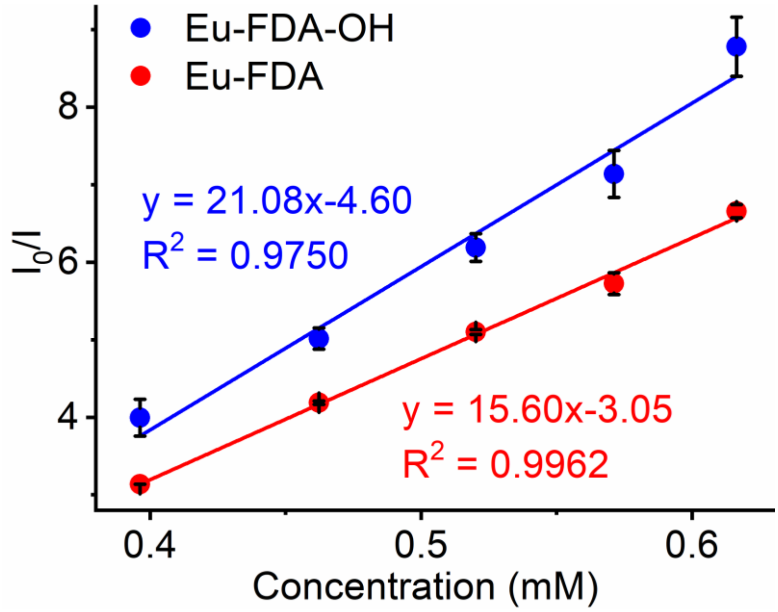

Figure S32. Intensity changes of Eu-FDA-OH and Eu-FDA towards additions of 1,2-diaminobenzene under the linear range.

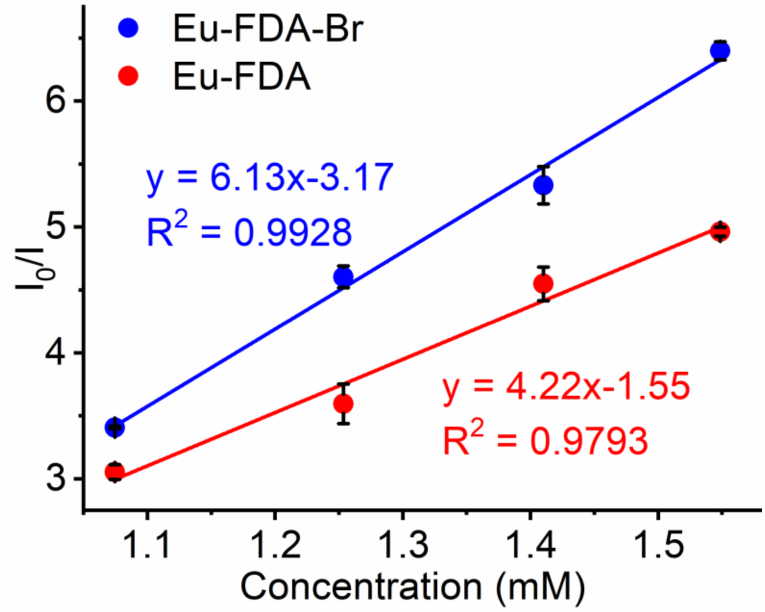

Figure S33. Intensity changes of Eu-FDA-Br and Eu-FDA towards additions of chlorothalonil under the linear range.

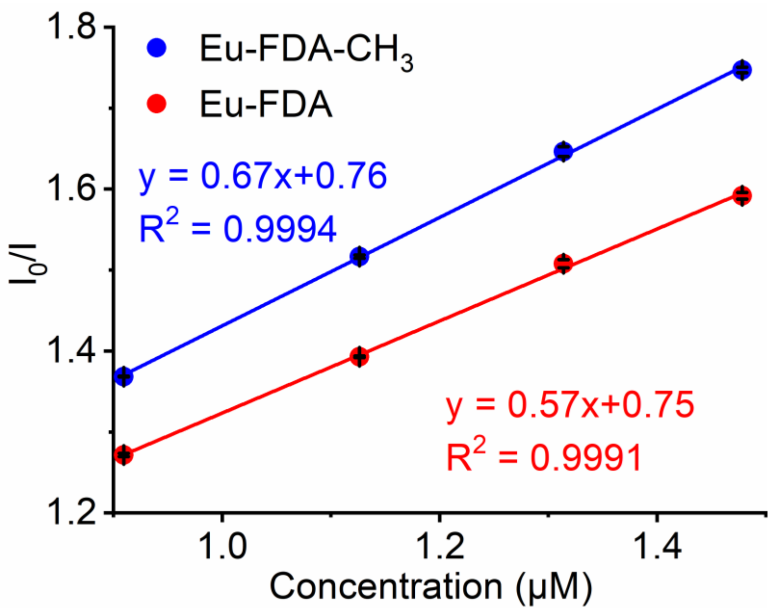

Figure S34. Intensity changes of Eu-FDA-CH_3 and Eu-FDA towards additions of resazurin under the linear range.

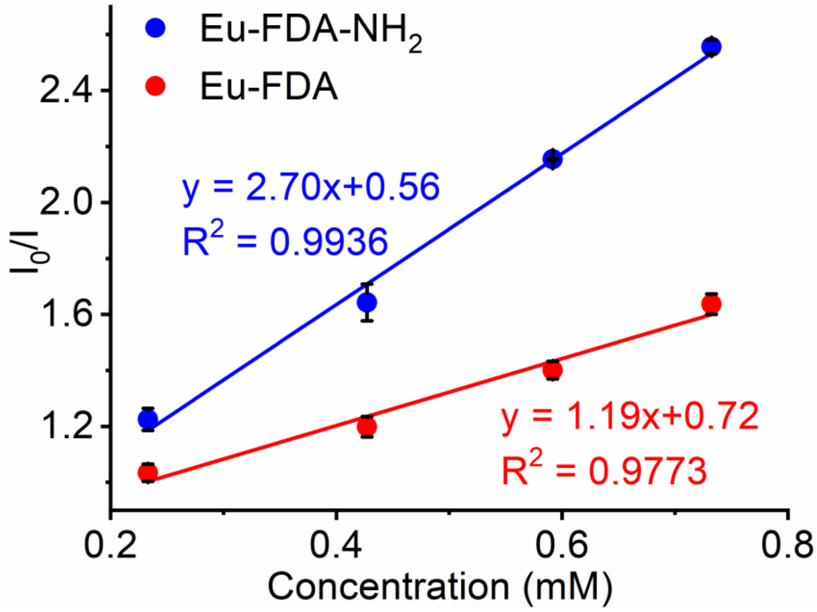

Figure S35. Intensity changes of Eu-FDA-NH_2 and Eu-FDA towards additions of dodecafluorosuberic acid under the linear range.

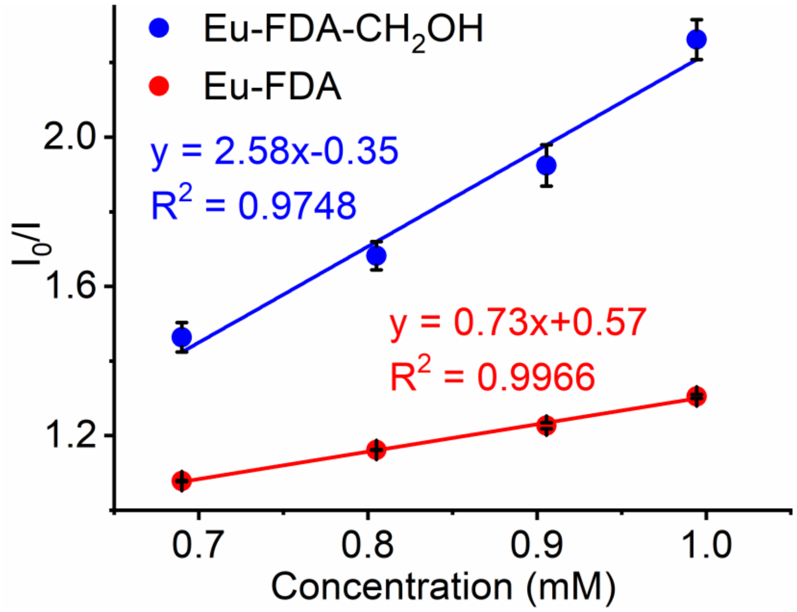

Figure S36. Intensity changes of Eu-FDA-CH_2OH and Eu-FDA towards additions of perfluorooctanoic acid under the linear range.

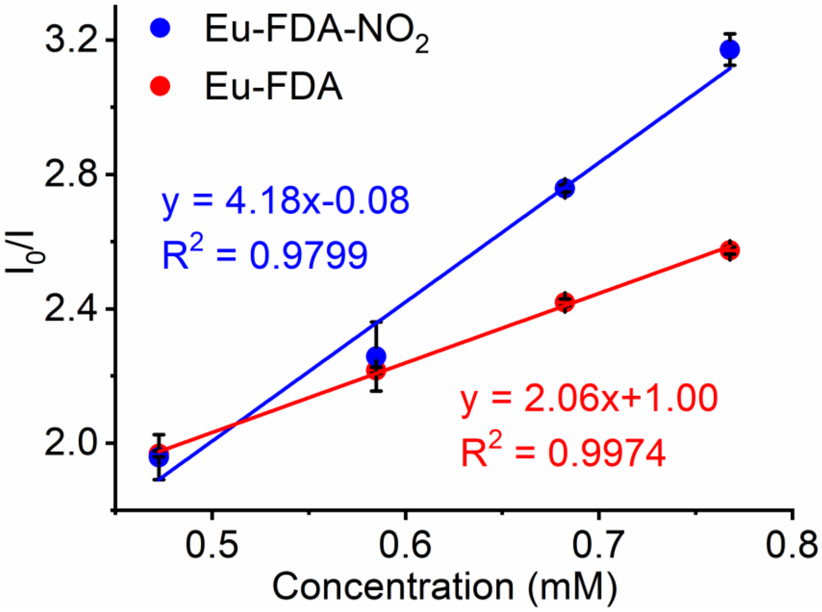

Figure S37. Intensity changes of Eu-FDA-NO_2 and Eu-FDA towards additions of nicotinamide under the linear range.

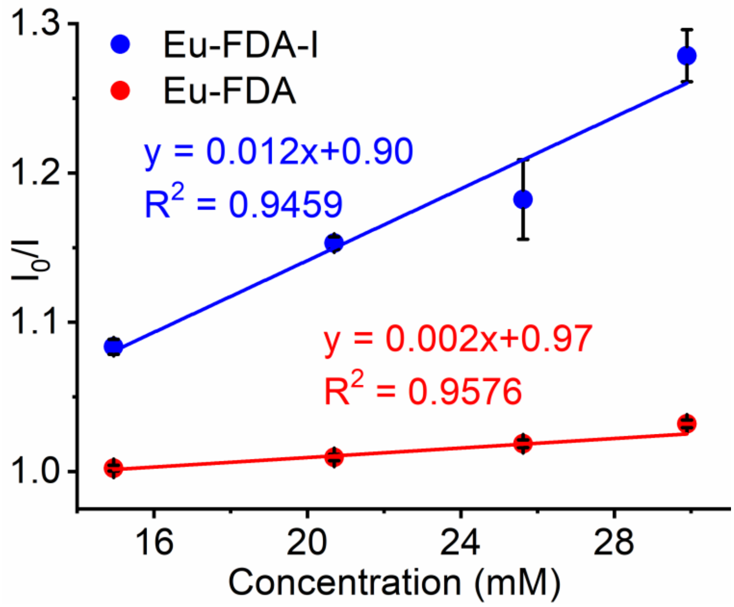

Figure S38. Intensity changes of Eu-FDA-I and Eu-FDA towards additions of trimethyl borate under the linear range.

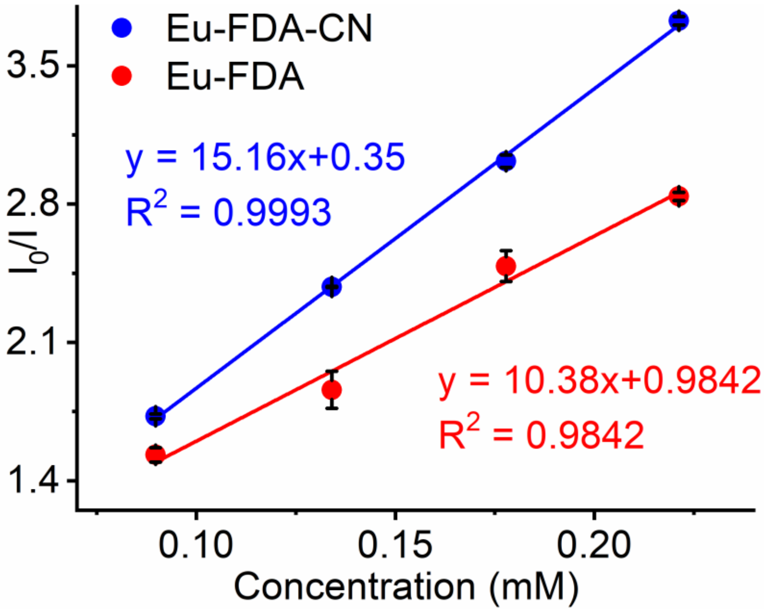

Figure S39. Intensity changes of Eu-FDA-CN and Eu-FDA towards additions of hydrogen peroxide under the linear range.

Sensing mechanisms

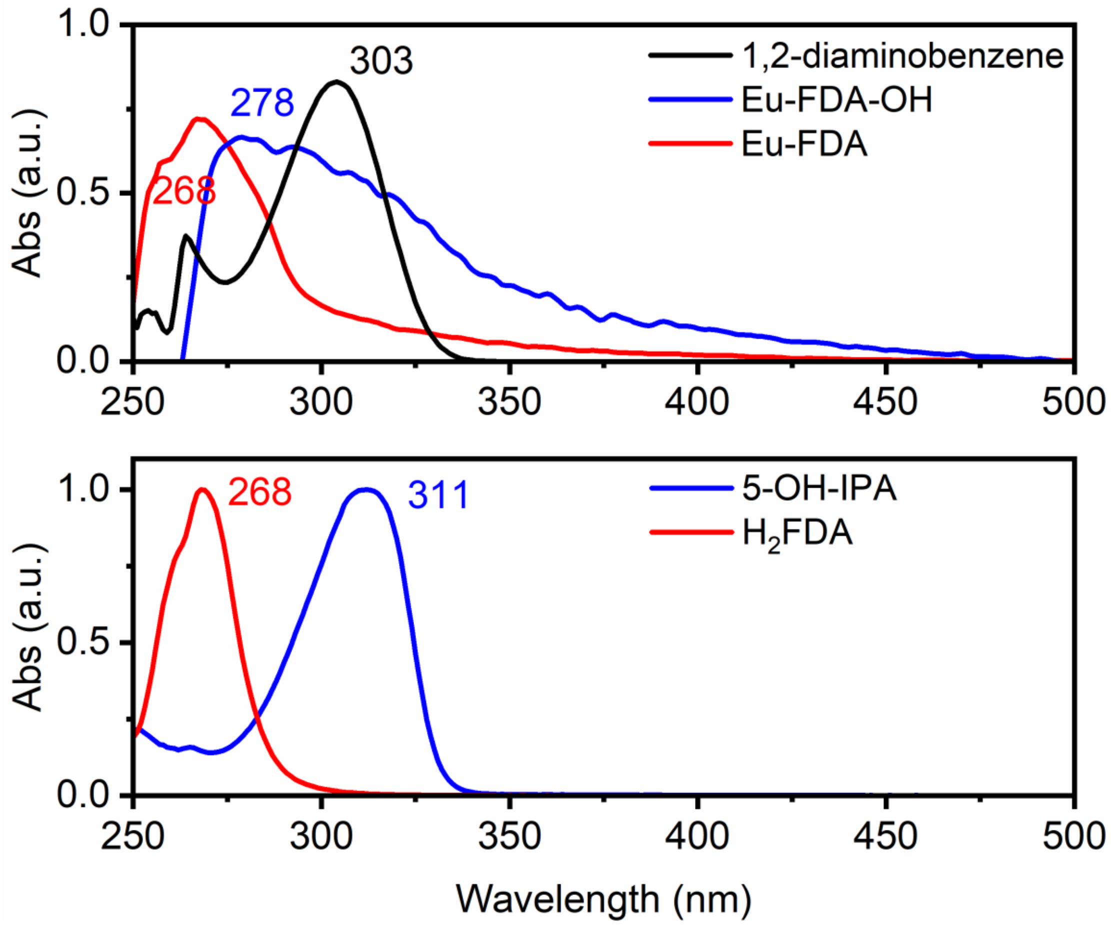

Figure S40. UV-vis spectra of 1,2-diaminobenzene, Eu-FDA-OH, Eu-FDA, 5-OH-IPA, and H_2FDA.

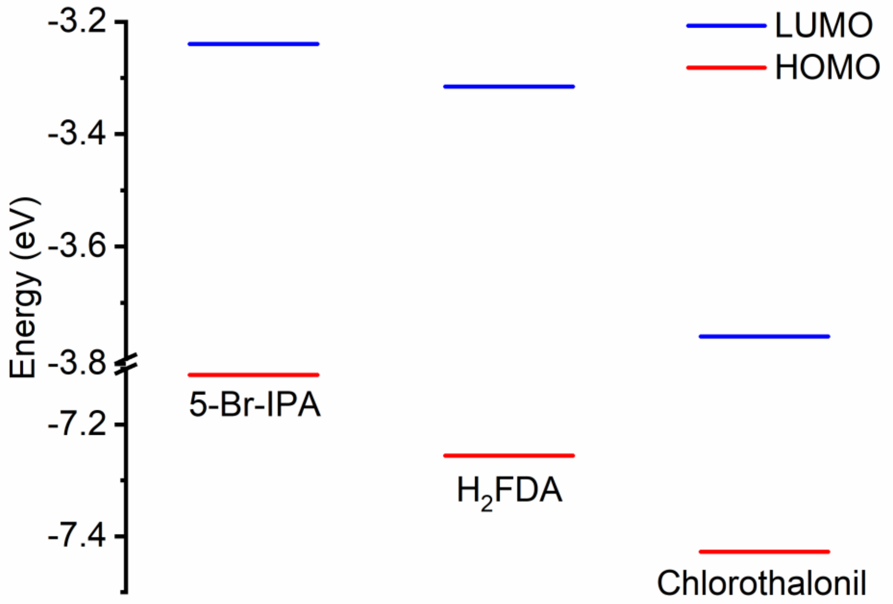

Figure S41. Energy levels of 5-Br-IPA, H_2FDA, and chlorothalonil.

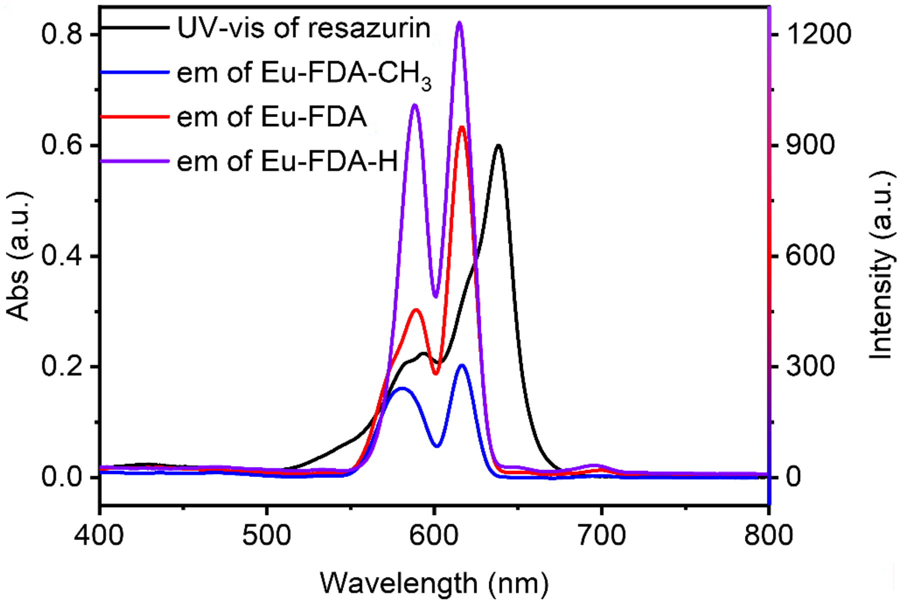

Figure S42. UV-vis spectrum of resazurin, and emission spectra of Eu-FDA-CH_3, Eu-FDA, and Eu-FDA-H.

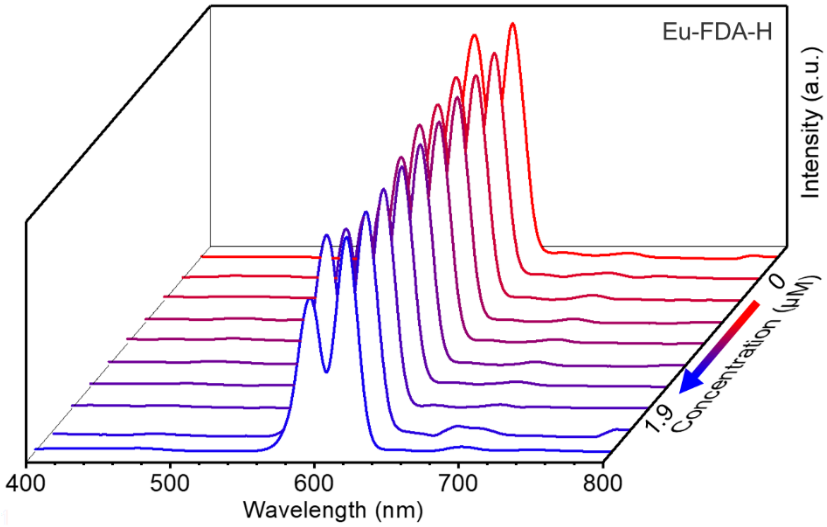

Figure S43. Emission spectra of Eu-FDA-H towards additions of resazurin.

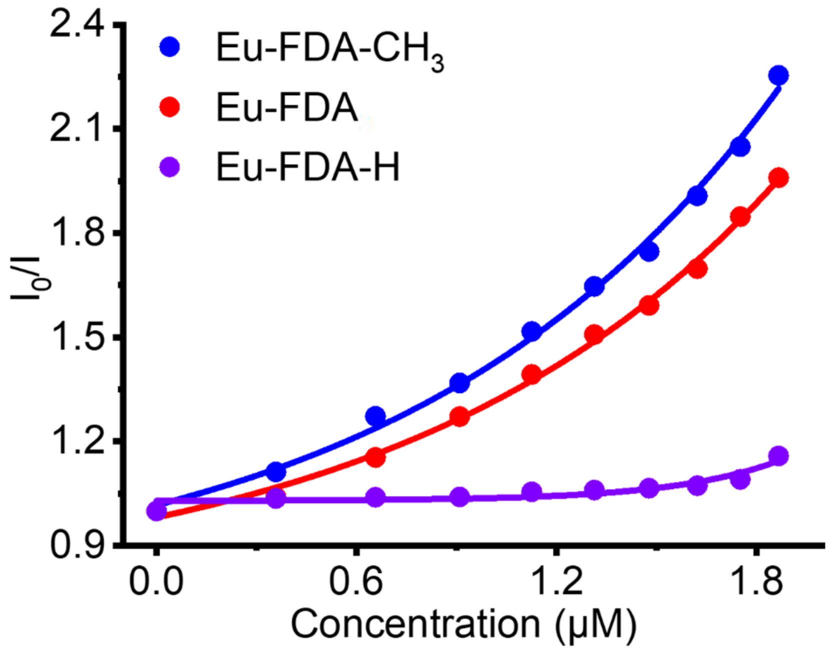

Figure S44. Emission intensity changes of Eu-FDA-CH_3, Eu-FDA, and Eu-FDA-H towards additions of resazurin.

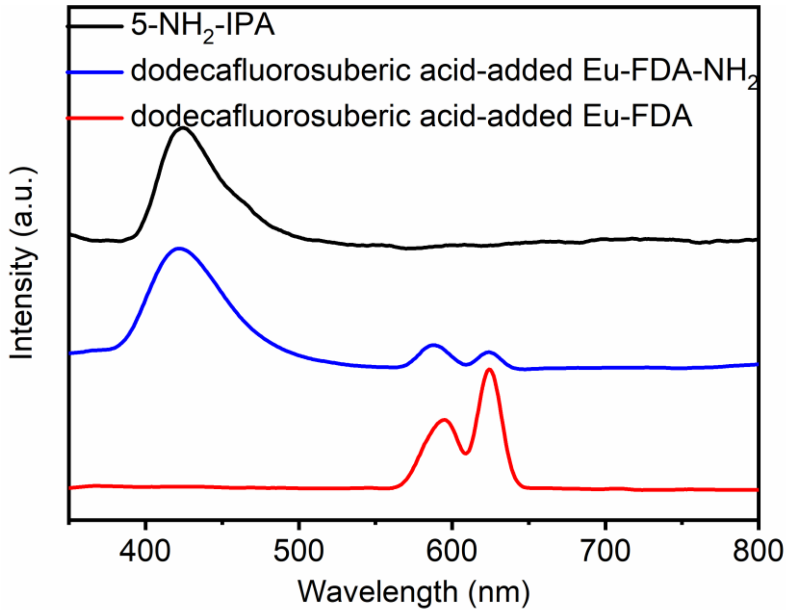

Figure S45. Emission spectra of 5-NH_2-IPA, dodecafluorosuberic acid-added Eu-FDA-NH_2, and dodecafluorosuberic acid-added Eu-FDA.

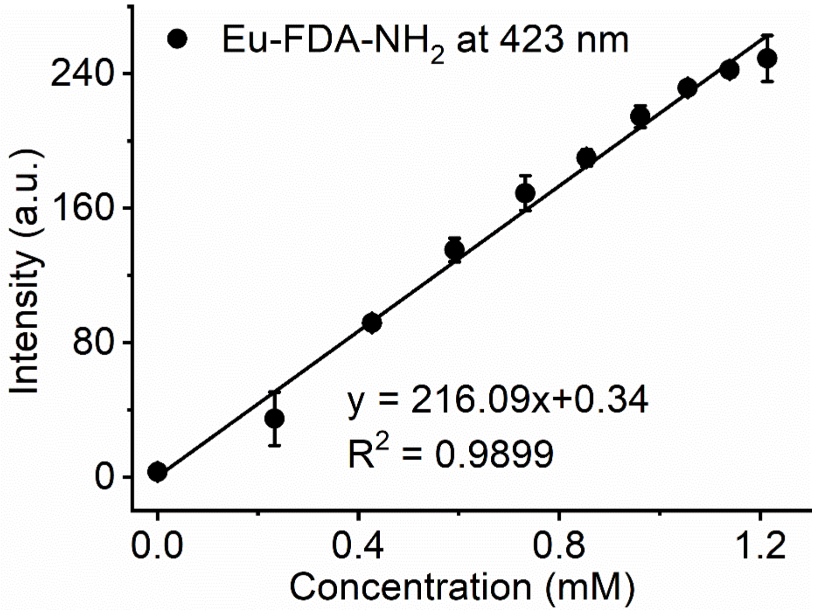

Figure S46. Intensity changes at 423 nm of Eu-FDA-NH_2 with the additions of dodecafluorosuberic acid.

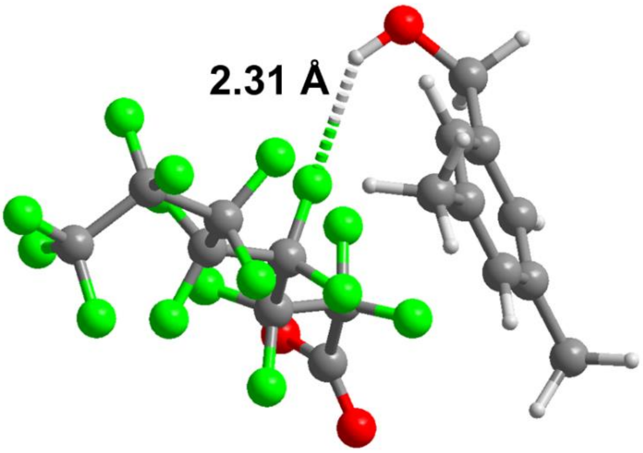

Figure S47. Molecule simulation of the binding modes of 5-CH_2OH-*m*-xylene and perfluorooctanoic acid. Atom code: C, grey; O, red; F, green; H, white.

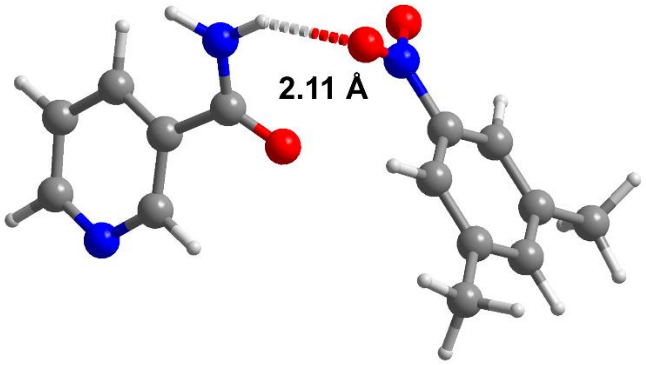

Figure S48. Molecule simulation of the binding modes of 5-NO_2-*m*-xylene and nicotinamide. Atom code: C, grey; O, red; N, blue; H, white.

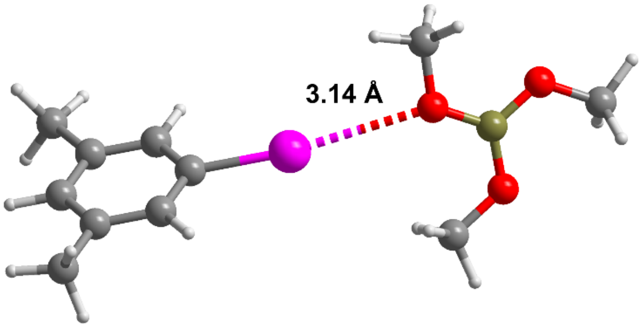

Figure S49. Molecule simulation of the binding modes of 5-I-*m*-xylene and trimethyl borate. Atom code: C, grey; O, red; I, pink; B, dark yellow; H, white.

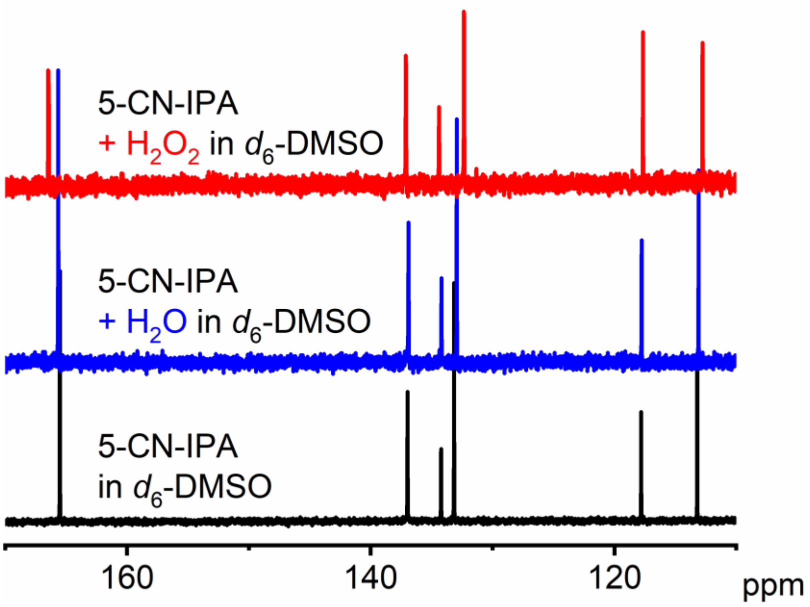

Figure S50. ^{13}C NMR spectra of 5-CN-IPA, H_2O-added 5-CN-IPA, and H_2O_2-added 5-CN-IPA.

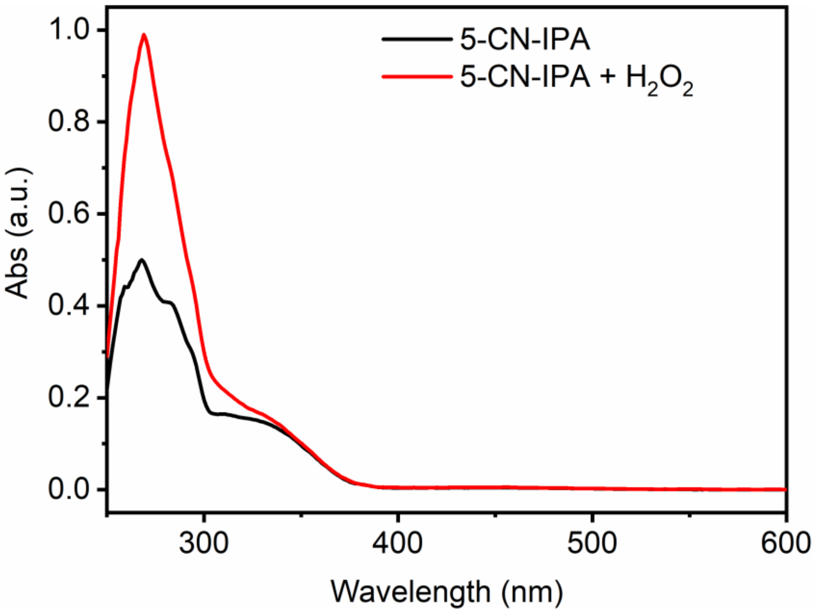

Figure S51. UV-vis spectra of 5-CN-IPA and H_2O_2-added 5-CN-IPA.

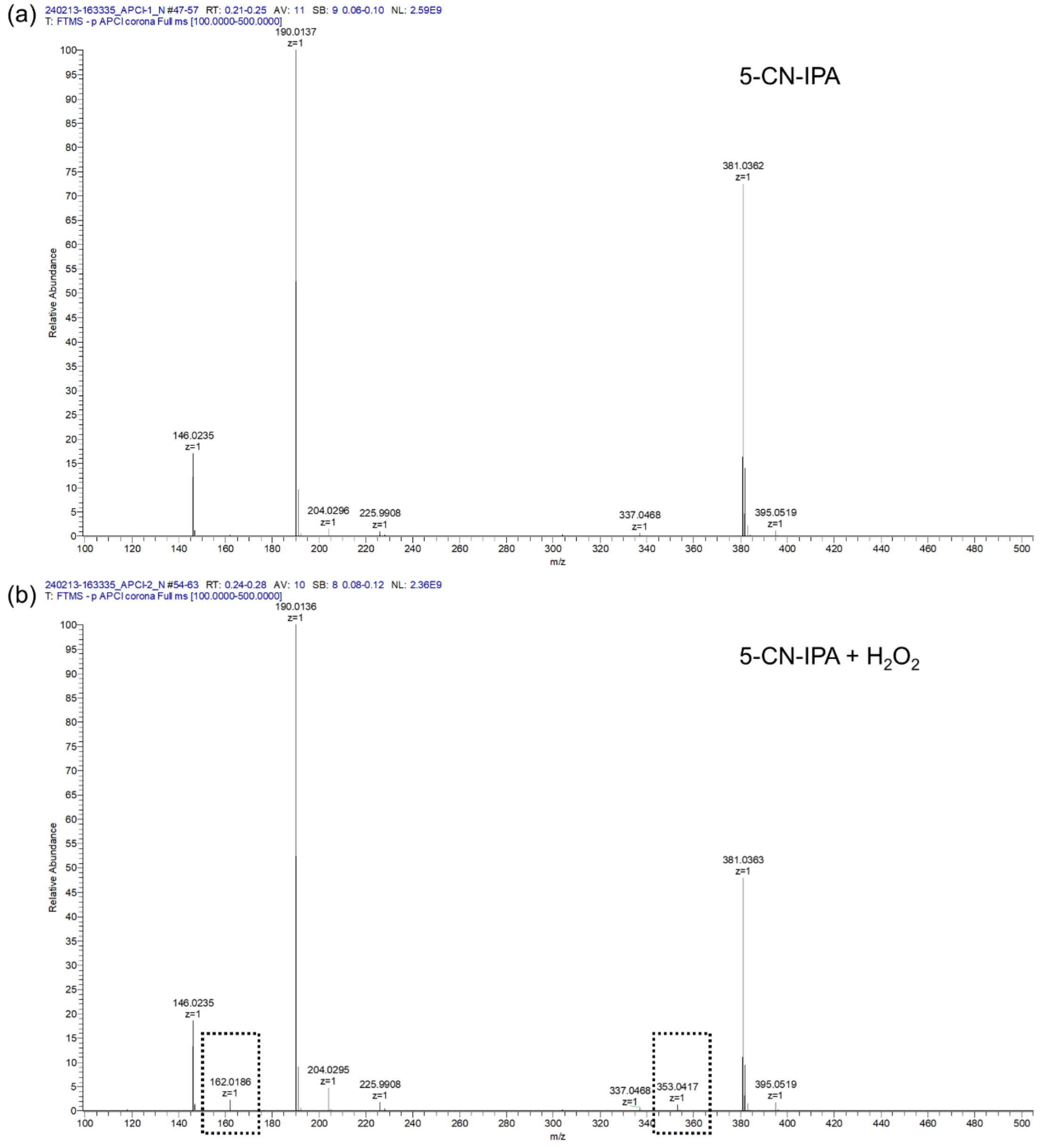

Figure S52. Mass spectra of 5-CN-IPA (a) and H_2O_2-added 5-CN-IPA (b).

Tables

Table S1. Crystallographic data and structure refinement details.

	**Eu-FDA**	**Eu-FDA-H**	**Eu-FDA-CH_3**	**Eu-FDA-Br**	**Eu-FDA-I**
CCDC	2328405	2328404	2328411	2328410	2328407
Formula	$C_{12}H_{10}EuNO_{8.5}$	$C_{13.8}H_{11.8}EuNO_{7.6}$	$C_{12.75}H_{10.25}EuNO_{8.25}$	$C_{13}H_{10.5}Br_{0.5}EuNO_8$	$C_{12.4}H_{7.2}EuI_{0.2}NO_{8.3}$
Formula wt	456.17	465.20	461.43	500.64	480.33
Temperature (K)	100.00(10)	100.00(10)	100.00(10)	100.00(10)	100.00(10)
Crystal System	orthorhombic	orthorhombic	orthorhombic	orthorhombic	orthorhombic
Space Group	*Pnma*	*Pnma*	*Pnma*	*Pnma*	*Pnma*
a (Å)	8.4118(3)	8.0977(2)	8.3468(1)	8.3434(2)	8.3193(2)
b (Å)	28.4552(9)	29.0122(5)	28.5686(4)	29.1069(5)	28.8713(6)
c (Å)	15.2921(6)	15.5447(2)	15.3367(2)	15.3587(2)	15.2886(3)
α (deg)	90	90	90	90	90
β (deg)	90	90	90	90	90
γ (deg)	90	90	90	90	90
Z	8	8	8	8	8
V (Å^3)	3660.3(2)	3651.95(12)	3657.13(8)	3729.87(12)	3672.15(14)
ρ_{calc} (g cm^{-3})	1.656	1.692	1.676	1.783	1.738
μ (mm^{-1})	24.881	24.914	24.901	25.670	27.440
F (000)	1760.0	1803.0	1782.0	1920.0	1829.0
R_{int}	0.0970	0.0742	0.1089	0.0646	0.0994
$^aR_1$, $^bwR_2$ ($I > 2\sigma(I)$)	0.0782, 0.1813	0.0621, 0.1601	0.0994, 0.2277	0.0760, 0.2053	0.1384, 0.3358
$^aR_1$, $^bwR_2$ (all data)	0.0910, 0.1900	0.0697, 0.1682	0.1046, 0.2319	0.0820, 0.2132	0.1472, 0.3422

$^aR_1 = \Sigma||F_o| - |F_c||/\Sigma|F_o|$, $^bwR_2 = [\Sigma w(F_o^2 - F_c^2)^2/\Sigma w(F_o^2)^2]^{1/2}$

	**Eu-FDA-NH$_2$**	**Eu-FDA-OH**	**Eu-FDA-NO$_2$**	**Eu-FDA-CN**	**Eu-FDA-CH$_2$OH**
CCDC	2328409	2328403	2328406	2328408	2328402
Formula	$C_{12.6}H_{10.3}EuN_{1.3}O_{8.2}$	$C_{12.6}H_{7.3}EuNO_{8.5}$	$C_{12.6}H_{10.3}EuN_{1.3}O_{8.8}$	$C_{13.8}H_{10.6}EuN_{1.6}O_{7.9}$	$C_{12.6}H_{11}EuNO_{8.5}$
Formula wt	463.08	460.65	472.68	477.20	464.38
Temperature (K)	100.00(10)	100.00(10)	100.00(10)	100.00(10)	100.00(10)
Crystal System	orthorhombic	orthorhombic	orthorhombic	orthorhombic	orthorhombic
Space Group	*Pnma*	*Pnma*	*Pnma*	*Pnma*	*Pnma*
a (Å)	8.3598(4)	8.2480(2)	8.3910(3)	8.2555(2)	8.3940(3)
b (Å)	28.3957(14)	28.5816(5)	28.8230(10)	29.1032(7)	28.6162(9)
c (Å)	15.4182(10)	15.4477(3)	15.3115(4)	15.4154(4)	15.3043(5)
α (deg)	90	90	90	90	90
β (deg)	90	90	90	90	90
γ (deg)	90	90	90	90	90
Z	8	8	8	8	8
V (Å^3)	3660.0(3)	3641.66(13)	3703.1(2)	3703.73(16)	3676.2(2)
ρ_{calc} (g cm^{-3})	1.681	1.680	1.696	1.712	1.678
μ (mm^{-1})	24.887	25.020	24.636	24.608	24.786
F (000)	1789.0	1767.0	1827.0	1846.0	1797.0
R_{int}	0.1446	0.0774	0.0972	0.0738	0.0901
$^aR_1$, $^bwR_2$ ($I > 2\sigma(I)$)	0.1285, 0.2889	0.0735, 0.1961	0.1120, 0.2866	0.0801, 0.2115	0.0903, 0.2402
$^aR_1$, $^bwR_2$ (all data)	0.1849, 0.3166	0.0823, 0.2069	0.1246, 0.3001	0.0912, 0.2258	0.0994, 0.2524

$^aR_1 = \Sigma||F_o| - |F_c||/\Sigma|F_o|$, $^bwR_2 = [\Sigma w(F_o^2 - F_c^2)^2/\Sigma w(F_o^2)^2]^{1/2}$

Table S2. Details of Eu-FDA series MOFs. The ratios represent the isophthalic acid derivatives : FDA.

	Feed ratio	Resulted ratio	Chemical formula
**Eu-FDA-H**	3:1	3:2	Eu(5-H-IPA)$_{0.9}$(FDA)$_{0.6}$(DMF)
**Eu-FDA-CH_3**	1:3	1:5	Eu(5-CH_3-IPA)$_{0.25}$(FDA)$_{1.25}$(DMF)
**Eu-FDA-Br**	1:1	1:2	Eu(5-Br-IPA)$_{0.5}$(FDA)(DMF)
**Eu-FDA-I**	1:3	2:13	Eu(5-I-IPA)$_{0.2}$(FDA)$_{1.3}$(DMF)
**Eu-FDA-NH_2**	1:2	1:4	Eu(5-NH_2-IPA)$_{0.3}$(FDA)$_{1.2}$(DMF)
**Eu-FDA-OH**	1:1	1:4	Eu(5-OH-IPA)$_{0.3}$(FDA)$_{1.2}$(DMF)
**Eu-FDA-NO_2**	1:3	1:4	Eu(5-NO_2-IPA)$_{0.3}$(FDA)$_{1.2}$(DMF)
**Eu-FDA-CN**	1:1	2:3	Eu(5-CN-IPA)$_{0.6}$(FDA)$_{0.9}$(DMF)
**Eu-FDA-CH_2OH**	1:2	2:13	Eu(5-CH_2OH-IPA)$_{0.2}$(FDA)$_{1.3}$(DMF)

Table S3. Selected luminescent sensing materials for analytes in literature.

Material	Analyte	K (M^{-1})	Literature
Eu-FDA-OH	1,2-diaminobenzene	2.11×10^{4}	This work
EuTb@NKU-102	1,2-diaminobenzene	3.60×10^{2}	10
Eu-FDA-Br	chlorothalonil	6.13×10^{3}	This work
β-CD	chlorothalonil	1.90×10^{3}	11
Eu-FDA-CH_3	resazurin	6.69×10^{5}	This work
Eu-FDA-NH_2	dodecafluorosuberic acid	2.70×10^{3}	This work
Eu-FDA-CH_2OH	perfluorooctanoic acid	2.58×10^{3}	This work
human serum albumin	perfluorooctanoic acid	1.33×10^{4}	12
MOF-76(Eu)	perfluorooctanoic acid	7.30×10^{2}	13
MOF-76(Tb)	perfluorooctanoic acid	1.20×10^{2}	13
Eu-FDA-NO_2	nicotinamide	4.18×10^{3}	This work
Eu-FDA-I	trimethyl borate	1.22×10	This work
Eu-FDA-CN	hydrogen peroxide	1.52×10^{4}	This work
$EuVO_4$	hydrogen peroxide	1.08×10	14
$CePO_4:Sm_x$	hydrogen peroxide	2.39×10^{3}	15
ZnOCPc-rGONS	hydrogen peroxide	3.20×10^{-2}	16
ZnOCPc-GONS	hydrogen peroxide	2.40×10^{-2}	16
$Ru(bpy)_3^{2+}$ in water	hydrogen peroxide	2.8	17
$Ru(bpy)_3^{2+}$ in GPS	hydrogen peroxide	2.8	17

References

(1) Sheldrick, G. M. Crystal structure refinement with SHELXL. *Acta Crystallogr., Sect. C: Struct. Chem.* **2015**, *71*, 3-8.

(2) Sheldrick, G. M. A short history of SHELX. *Acta Crystallogr., Sect. A: Found. Crystallogr.* **2008**, *64*, 112-122.

(3) Zhou, J.; Li, H.; Zhang, H.; Li, H.; Shi, W.; Cheng, P. A bimetallic lanthanide metal–organic material as a self-calibrating color-gradient luminescent sensor. *Adv. Mater*. **2015**, *27*, 7072-7077.

(4) Neese, F.; Wennmohs, F.; Becker, U.; Riplinger, C. The ORCA quantum chemistry program package. *J. Chem. Phys.* **2020**, *152*, 224108.

(5) Neese, F. Software update: The ORCA program system—Version 5.0. *WIREs Comput. Mol. Sci.* **2022**, *12*, e1606.

(6) Grimme, S.; Hansen, A.; Ehlert, S.; Mewes, J.-M. r2SCAN-3c: A "Swiss army knife" composite electronic-structure method. *J. Chem. Phys.* **2021**, *154*, 064103.

(7) Kruse, H.; Grimme, S. A geometrical correction for the inter- and intra-molecular basis set superposition error in Hartree-Fock and density functional theory calculations for large systems. *J. Chem. Phys.* **2012**, *136*, 154101.

(8) Caldeweyher, E.; Bannwarth, C.; Grimme, S. Extension of the D3 dispersion coefficient model. *J. Chem. Phys.* **2017**, *147*, 034112.

(9) Caldeweyher, E.; Ehlert, S.; Hansen, A.; Neugebauer, H.; Spicher, S.; Bannwarth, C.; Grimme, S. A generally applicable atomic-charge dependent London dispersion correction. *J. Chem. Phys.* **2019**, *150*, 154122.

(10) Han, Z.; Wang, K.; Chen, Y.; Li, J.; Teat, S. J.; Yang, S.; Shi, W.; Cheng, P. A multicenter metal-organic framework for quantitative detection of multicomponent organic mixtures. *CCS Chem.* **2022**, *4*, 3238-3245.

(11) Ren, H.; Li, G.; Yue, X.; Chen, X.; Zhang, C.; Wang, Z.; Peng, C. Highly sensitive fluorescent turn-on lateral flow strip for chlorothalonil based on an indicator displacement ratiometric fluorescent assay. *Sens. Actuators B Chem*. **2023**, *381*, 133414.

(12) Chen, H.; Wang, Q.; Cai, Y.; Yuan, R.; Wang, F.; Zhou, B. Investigation of the interaction mechanism of perfluoroalkyl carboxylic acids with human serum albumin by spectroscopic methods. *Int. J. Environ. Res. Public Health* **2020**, *17*, 1319.

(13) Song, M.; Yu, R.; Shang, Y.; Tashpulatov, K.; Sun, H.; Zeng, J. Lanthanide metal-organic frameworks as ratiometric fluorescent probes for real-time monitoring of PFOA photocatalytic degradation process. *Chemosphere* **2024**, *363*, 142946.

(14) Duée, N.; Ambard, C.; Pereira, F.; Portehault, D.; Viana, B.; Vallé, K.; Autissier, D.; Sanchez, C. New synthesis strategies for luminescent YVO_4:Eu and $EuVO_4$ nanoparticles with H_2O_2 selective sensing properties. *Chem. Mater.* **2015**, *27*, 5198-5205.

(15) Vinothkumar, G.; Arun, I. L.; Arunkumar, P.; Ahmed, W.; Ryu, S.; Cha, S. W.; Babu, K. S. Structure dependent luminescence, peroxidase mimetic and hydrogen peroxide sensing of samarium doped cerium phosphate nanorods. *J. Mater. Chem. B* **2018**, *6*, 6559-6571.

(16) Shumba, M.; Mashazi, P.; Nyokong, T. "Turn on" fluorescence enhancement of Zn octacarboxyphthaloyanine-graphene oxide conjugates by hydrogen peroxide. *J. Lumin.* **2016**, *170*, 317-324.

(17) Mills, A.; Tommons, C.; Bailey, R. T.; Tedford, M. C.; Crilly, P. J. Reversible, fluorescence-based optical sensor for hydrogen peroxide. *Analyst* **2007**, *132*, 566-571.
